# Human Health Risks of Conducted Electrical Weapon Exposure: A Systematic Review

**DOI:** 10.1001/jamanetworkopen.2020.37209

**Published:** 2021-02-12

**Authors:** Christos Baliatsas, Jenny Gerbecks, Michel L. A. Dückers, C. Joris Yzermans

**Affiliations:** 1 Netherlands Institute for Health Services Research (Nivel), Utrecht, the Netherlands; 2 ARQ National Psychotrauma Centre, Diemen, the Netherlands; 3 University of Groningen, Groningen, the Netherlands

## Abstract

**Importance:**

Conducted electrical weapons (CEWs) are used broadly as a less-lethal force option for police officers. However, there is no clear picture of the possible health risks in humans on the basis of rigorously assessed scientific evidence from the international peer-reviewed literature.

**Objective:**

To synthesize and systematically evaluate the strength of published evidence for an association between exposure to different models of CEWs and adverse acute as well as chronic conditions.

**Evidence Review:**

Following a preregistered review protocol, the literature search strategy was based on a search of reviews published between January 1, 2000, and April 24, 2020, of PubMed, MEDLINE, EMBASE, Web of Science, PsycINFO, and Cochrane Library, as well as relevant online databases and bibliographic sources, such as reference sections of recent publications. The identified studies were independently assessed in terms of scope, relevance, methodologic bias, and quality. Peer-reviewed publications of human studies were included, using original data and with a focus on the use of taser CEWs in the context of law enforcement. Eligible studies examined clearly defined health outcomes as dependent variables following exposure to a CEW. The review followed the relevant sections of the Preferred Reporting Items for Systematic Reviews and Meta-analyses reporting guideline. A meta-analysis could not be conducted.

**Findings:**

Of the 1081 unique records screened, 33 relevant studies were identified, all of them of experimental design and conducted in the US. Eleven studies had a low risk of bias and 22 had a higher bias risk. Studies focused on outcomes such as physiologic stress responses, heart rate, blood pressure, arrhythmias, or cognitive performance. Independently of bias risk, the studies reported few or no acute health problems, apart from the wounds caused by the darts. Furthermore, no long-term outcomes were studied. Most of the studies were performed on healthy, physically fit individuals (eg, police officers) in a controlled setting, with short exposure duration (5 seconds). Half of the studies, mainly those with a higher risk of bias, were at least partly funded by the manufacturer.

**Conclusions and Relevance:**

Based on the findings of the reviewed studies, the risk for adverse health outcomes due to CEW exposure can be currently estimated as low. However, most of the reviewed studies had methodologic limitations. Considering that recruited participants were not representative of the population that usually encounters a CEW deployment, it is not possible to draw conclusions regarding exposure outcomes in potentially vulnerable populations or high-risk groups, such as those under the influence of substances.

## Key Points

**Question:**

What are the health risks associated with exposure to an electrical weapon?

**Findings:**

This systematic review of 33 studies on use of conducted electrical weapons found no evidence that electrical weapon exposure is associated with adverse health outcomes. However, most of the existing studies recruited healthy and physically fit individuals and had important methodologic limitations.

**Meaning:**

It is unlikely that the existing evidence constitutes a good representation of real-life field situations.

## Teaser

This systematic review describes studies on the reactions of persons exposed to tasers in law enforcement.

## Introduction

Given that police personnel must regularly deal with dangerous and often life-threatening situations, availability of appropriate equipment is important. The use of firearms comes with risks for the offenders as well as the police officers and in some cases for bystanders. In recent years, there has been a shift to alternative, less-lethal law enforcement options, such as conducted electrical weapons (CEWs), pepper spray, and batons. In this article, the focus is on the taser, a type of CEW.^1^ Over the past 2 decades, the taser CEW has been considered a means of law enforcement and especially self-defense during encounters with aggressive or violent individuals, particularly those who may not respond to alternatives to the use of force.^2,3^ The term *taser* (Thomas A. Swift electronic rifle) refers to a specific type of CEW, produced by Axon Enterprise Inc (formerly TASER International Inc).^1,4^ The taser CEW was developed in the US and is currently, as in many other countries, part of basic police equipment.^4,5^ Several types of taser CEWs exist (eTable 1 in the Supplement) as do different deployment methods. Such CEWs can be used in 2 ways: either by firing a barbed dart or by using the stun mode. The first option produces an electric current that causes muscle contraction, leading to a temporary incapacitation of the victim, and the second option provokes physical discomfort/pain.^1,4,5^

Currently, CEWs are used broadly as a less-lethal force option for police, but their use has been controversial from the early stages of production and their safety has been a subject of debate in both the media^6,7^ and scientific community.^8^ Despite the increasing availability of CEWs within the police force internationally, there is no clear picture of possible adverse health outcomes on the basis of rigorously assessed scientific evidence. Axon Enterprise Inc has published guidelines regarding taser CEW use,^9^ suggesting that exposure is safe for healthy people who are not under the influence of substances, are not pregnant, are not children or older individuals, and have no psychiatric disorders, and if the exposure lasts no longer than a few seconds and only in certain parts of the body. However, to date, most of the existing literature has focused on the technical aspects of CEWs, anecdotal or non–peer-reviewed reports, or animal studies.

To our knowledge, scientific evidence from the international peer-reviewed literature on the potential health effects of exposure to CEWs in humans has not been systematically reviewed following rigorous and replicable methods. Our systematic review aimed to fill this gap in the literature by identifying relevant studies conducted in the past 20 years to synthesize and systematically assess the strength of evidence for an association between exposure to different models of taser CEWs and acute as well as chronic adverse conditions. In addition, this review examined whether any possible risk groups can be distinguished based on the current evidence.

## Methods

### Primary Search Strategy

This study followed the relevant sections of the Preferred Reporting Items for Systematic Reviews and Meta-analyses (PRISMA) reporting guideline for systematic reviews.^10^ The study methodology protocol has been preregistered in the PROSPERO platform.^11^ A meta-analysis was not conducted because of high heterogeneity among the studies.

A wide range of relevant search terms regarding exposure to CEWs was used to form the search strategy (eg, *taser*, *Axon*, *electrical weapon*, and *electric shock weapon*). Before the definitive strategy and search term selection were developed, a pilot search was conducted on PubMed and MEDLINE where the number and applicability of various search terms were tested. The detailed search per main database is included in eAppendix 6 in the Supplement. As a first step, we made an inventory of key publications, such as reviews, based on a pilot search on PubMed and MEDLINE. Second, we developed our literature research strategy protocol on the basis of relevant search terms from the identified articles and exchange of feedback within the project team. The search strategy was based on an extensive literature search in the following major databases: PubMed, including all records of MEDLINE; Web of Science; PsycINFO; EMBASE; and Cochrane Central Register of Controlled Trials.

In addition, a basic search was conducted using the following relevant online databases: ClinicalTrials.gov register, European Union (EU) Clinical Trials register, US National Institute of Justice database, and the CEPOL (EU Agency for Law Enforcement Training) database. Furthermore, the reference sections of several articles were screened to check whether any relevant studies were missed in the primary and additional searches.

### Selection Criteria

Several major inclusion and exclusion criteria were established a priori. Studies were included by 3 of us (C.B., J.G., and C.J.Y.) if the following criteria were met: published or accepted for publication between January 1, 2000, and April 24, 2020; primary scientific studies including original data; published in English, Dutch, French, German, or Spanish; published in peer-reviewed journals; had explicit focus on the use of taser CEWs in the context of law enforcement; assessed clearly defined (self-reported or physician-diagnosed/objectively assessed) health symptoms and conditions, health status or symptom scores, and/or physiologic measures as dependent variables; included only humans; and focused on CEWs as the exposure source (only models from taser and Axon Enterprise Inc, in particular, M26, X26[P], X3, X2, XREP, and/or more recent models). Studies focusing on equipment/devices that did not fall within this classification, such as stun guns, batons/prods, and belts, were excluded.

There was no restriction in terms of demographic characteristics or investigated health outcomes. Purely descriptive studies in which no clear exposure-outcome associations were tested, reviews, and case studies were excluded. Additional details are presented in eTable 2 in the Supplement.

### Assessment 

Given that the risk of methodologic bias and study and reporting quality are not synonymous,^12^ both the risk of bias and general study quality were independently assessed by 2 of us (C.B. and J.G.). After consensus and pilot testing within the research team, a modified version of the AXIS (Appraisal tool for Cross-Sectional Studies) quality assessment tool^13,14^ was used so as to be applicable to different study designs^15^ (eAppendix 1 in the Supplement).

Bias risk was based on 3 items of the AXIS tool related to 3 fundamental methodologic aspects: statistical power, sampling, and statistical methods and replicability (numbers 3 [Was the sample size justified?], 4 [Was the target population clearly defined with appropriate population base/unbiased sampling?], and 6 [Were methods, including statistical methods, sufficiently described to enable them to be repeated?]). A study was classified as having a high risk of bias if it scored 0 on at least 2 of those 3 items. For the studies with a lower risk of bias, hierarchy of evidence based on study design was also documented following the Quality Rating Scheme for Studies and Other Evidence^16^: a modified classification was applied as follows: higher evidence quality (properly conducted randomized clinical trials, controlled trials without randomization, or prospective, comparative cohort trials) and lower evidence quality (nonrandomized, uncontrolled experimental studies or observational studies).

Regarding general study quality, a score was generated based on all items of the modified AXIS tool (maximum score, 10, 11, or 12, depending on the study design) and divided into 3 categories: low, moderate, and high. The involvement of sponsors was evaluated separately by documenting the funding source for each of the included studies. Assessment of risk of bias and study quality was performed a posteriori and was not a prerequisite for considering a study as eligible for the review.

### Procedure

During the first phase of the screening, duplicate records from the pool of identified studies were excluded. The members of the project team independently screened the titles and abstracts of the identified studies using the selection protocol formed on the basis of the aforementioned broader inclusion and exclusion criteria. Studies that clearly did not meet the inclusion criteria at this stage of the screening process were excluded. Possible eligible studies underwent a full-text assessment.

During the second phase of the screening, the potentially relevant studies were assessed by 2 of us (C.B. and J.G.). Discrepancies in terms of scope or relevance of a particular article during the screening were resolved by the involvement of a third reader (C.J.Y.) and discussion among the project team members until consensus was reached. For a third of the studies screened for relevance by both researchers, interrater reliability was calculated based on Cohen κ assessment for binary assessments.^17,18^ The value of the Cohen κ indicated good agreement (87%, κ = 0.75).

In the third phase, bias evaluation and general quality assessment were carried out independently by 2 of us (C.B. and J.G.) for all relevant studies, with raters being blind to each other's assessment. Interrater reliability was good (good agreement at 88%, κ = 0.75) and discrepancies between assessments were resolved by the involvement of a third researcher (C.J.Y.). Regarding general quality assessment, interrater reliability was evaluated for a third of all included studies, reaching agreement in all classifications after discussion about minor differences in assessments.

The selection process was documented in a PRISMA flowchart.^10^ For the extraction of data, we used a form that was tested on a selection of 10 relevant studies. After agreement was reached on the form, the data were extracted, coded, and imported into tables and the accuracy of the extraction was checked. Differences in assessment or interpretation were resolved through consultation among us. The following characteristics were extracted for each study: authors of the article, year of publication, country in which the study was conducted, CEW model and exposure duration, sample size and type, study design, general study quality, main findings, and funding source.

## Results

### Literature Search and Study Characteristics

The PRISMA flowchart (Figure) shows the literature search process. Of the 1081 unique records screened, 362 potentially relevant studies underwent full-text assessment, and 329 studies were excluded. During this stage of the search, we identified and documented 16 solely descriptive studies on the topic (eAppendix 2 in the Supplement), 60 case studies (eAppendix 3 in the Supplement), and 30 review or overview papers (eAppendix 4 in the Supplement). As a result of this process, 33 research articles from the peer-reviewed literature were included in this review.

**Figure.**
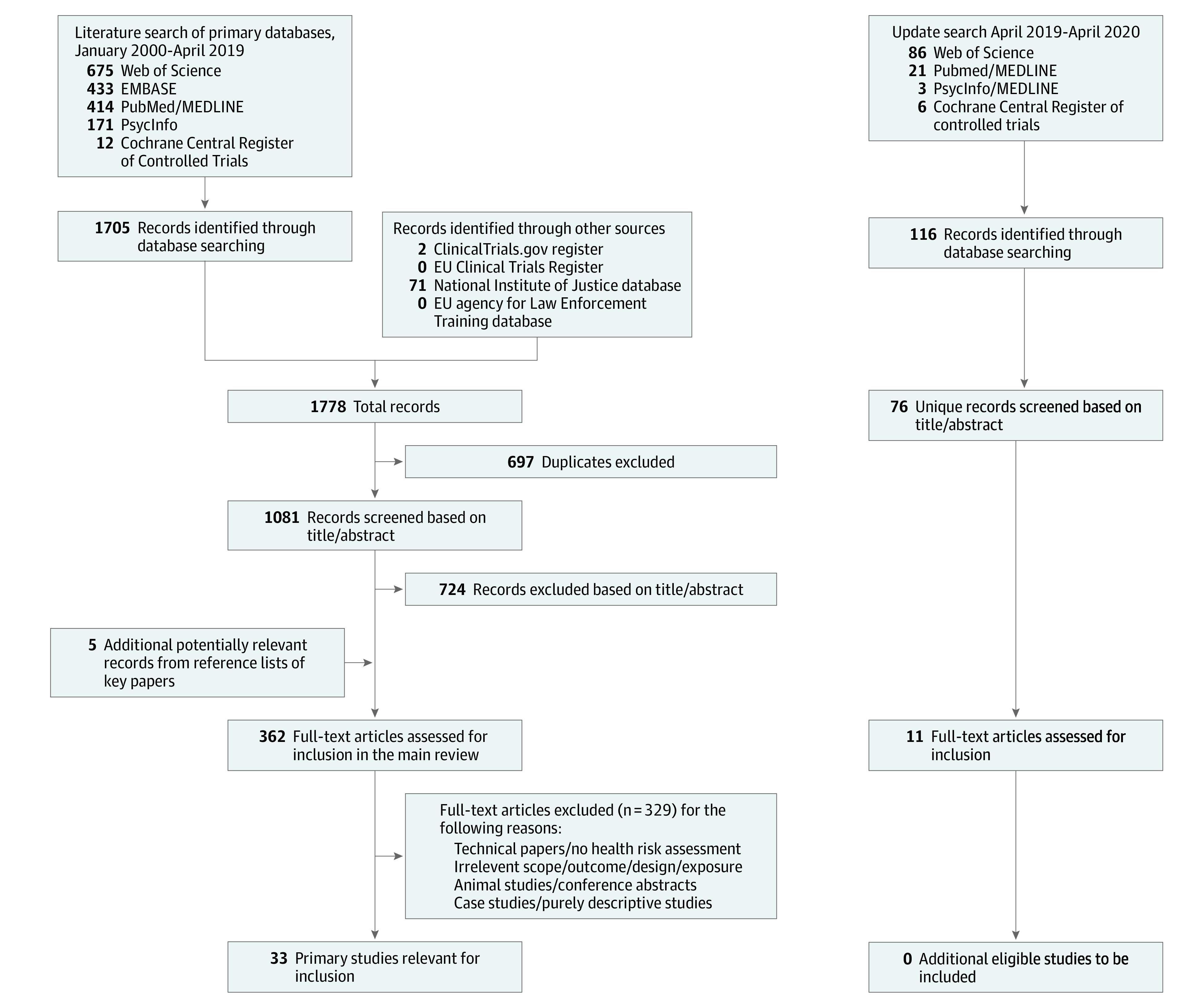
Flowchart Illustrating the Study Selection Process EU indicates European Union.

Most of the studies (n = 29) were nonrandomized trials (namely provocation studies with or without a control group), while 4 were randomized clinical trials. Most of the studies (26 [79%]) were conducted in the US and focused on the taser X26 as the exposure source. Twenty-seven studies reported that they involved law enforcement officers as participants, sometimes together with medical personnel or civilians (taser CEW trainees), and 19 studies (58%) recruited less than 30 participants. In 30 studies (91%), the maximum exposure duration to the CEW current was 15 seconds (up to 5 seconds in 19 studies), and the darts were often replaced by sprung metal clips (alligator clips). In one study the duration reached 30 seconds. Most of the investigated outcomes concerned physiologic measures of different aspects of the person’s psychophysiologic state, such as heart rate, blood pressure, cognitive skills, and conditions such as cardiac arrhythmias and acidosis.

In terms of general quality assessment, 6 studies (18%) scored high, 25 (76%) had a moderate score, and 2 (6%) had a low score. Eleven studies were assessed as having a low risk of bias regarding statistical power, sampling, and statistical methods and replicability. The design of 4 of those studies (12%) referred to a higher quality of evidence (randomized clinical trial and controlled studies). Seventeen studies were at least partly funded by the manufacturer (Axon Enterprises Inc). In 4 studies the funding source was not reported. In addition, the sample sizes of studies receiving funding from the CEW manufacturer appeared to be consistently lower.

### Association Between CEW Exposure and Outcomes

Independently of the general quality of the included studies, findings were categorized based on the possible risk of methodologic bias (Table 1^19,20,21,22,23,24,25,26,27,28,29^ and Table 2^30,31,32,33,34,35,36,37,38,39,40,41,42,43,44,45,46,47,48,49,50,51^). Table 1 presents information on the 11 included studies that were found to have a lower possible risk of bias. These studies generally showed few or no acute health outcomes other than superficial wounds caused by the darts in some cases. Except for sporadically observed increases in blood pressure, no significant changes occurred in heart rhythm and physiologic stressors, such as markers of acidosis, following prolonged exposure. Two studies assessing cognitive skills showed contradictory findings.^29,36^ The study with the smallest sample (n = 4) looked at possible adverse outcomes associated with CEW exposure on people with a pacemaker and reported no significant alterations in heart rate or blood pressure, or any interference with the implanted device.^24^

**Table 1.** Results of Included Studies With a Lower Risk of Bias

Source	CEW model, exposure duration	Sample (No.)	Study design	General study quality	Main findings	Funding source
Vilke et al,^19^ 2007, US	X26, 5 s	Law enforcement officers (32)	Nonrandomized trial (no control group)	High	CEW exposure was not associated with clinically significant changes in physiologic stress. All changes in physiologic measurements returned to baseline levels within 30 min.	National Institute of Justice
Vilke et al,^20^ 2008, US	X26, 1-5 s	Law enforcement officers (32)	Nonrandomized trial (no control group)	Moderate	There were no clinically significant changes in pre- and postmeasurement ECGs. No cardiac dysrhythmias or interval or morphologic changes were found.	Not reported
Vilke et al,^21^ 2009, US	X26, 5 s	Law enforcement officers (25)	Nonrandomized trial (including a control comparison)	Moderate	CEW exposure after vigorous exercise was not associated with clinically significant changes in ventilator or blood parameters of physiologic stress.	National Institute of Justice
VanMeenen et al,^22^ 2010, US	X26, 5, 4, 3 or 2 s	Law enforcement trainees (118)	Nonrandomized trial (no control group)	High	There was no evidence of an association between CEW exposure and direct injury to cardiac or skeletal muscle tissue.	National Institute of Justice
Kane and White,^23^ 2016, US	Not reported	Adult students (73)	Randomized clinical trial	High	CEW exposure led to (short) reductions (<60 min) in verbal learning ability and memory.	National Institute of Justice
Stopyra et al,^24^ 2017, US	X26, 5 s	Patients with a pacemaker or ICD (4)	Provocation (pilot) study, no control group	Moderate	No CEW-associated dysrhythmias were observed; however, all ICD devices interpreted the cyclical CEW discharge as ventricular tachycardia, but no shock therapy was given due to cessation of the tachycardia sensing after 5 s.	National Institute of Justice
Kroll et al,^25^ 2018, US	X26, 5 s	Law enforcement officers (31)	Nonrandomized trial (no control group)	High	No significant increase in serum serotonin levels was associated with exposure, indicating no increased risk for CEW-induced excited delirium.	US Joint Non-Lethal Weapons Program
Ho et al,^26^ 2009, US	X26, 15 s	Law enforcement officers and medical personnel (38)	Nonrandomized trial (no control group)	Moderate	Prolonged CEW exposure on physically exhausted humans was not associated with changes in acidosis, hyperkalemia, or cardiac injury.	Axon Enterprise Inc
Ho et al,^27^ 2010, US	X26, 10 s	Law enforcement officers (12)	Nonrandomized trial (with a control group)	Moderate	CEW exposure was not associated with acidosis. Smaller changes in markers of acidosis were observed during exposure vs control conditions, such as physical resistance/activity tasks.	Axon Enterprise Inc
Ho et al,^28^ 2011, US	X26, 15 s	Law enforcement officers and medical personnel (25)	Nonrandomized trial (no control group)	Moderate	Prolonged duration of CEW exposure in exhausted humans was not associated with detectable changes in ECGs.	Axon Enterprise Inc
Dawes et al,^29^ 2018, US	X2, 5 s	Law enforcement officers and citizens (24)	Nonrandomized trial (with a control group)	Moderate	No significant neurocognitive performance decrements were observed after CEW exposure.	Axon Enterprise Inc

Abbreviations: CEW, conducted electrical weapon; ECG, electrocardiogram; ICD, implantable cardioverter defibrillator.

**Table 2.** Results of Included Studies With a Higher Risk of Bias

Source	CEW model, exposure duration	Sample type (No.)	Study design	General study quality	Main findings	Funding source
Levine et al,^30^ 2007, US	X26, 3 s (on average)	Police officers (105)	Nonrandomized trial (no control group)	Moderate	CEW exposure was associated with a significant increase in heart rate, but no other cardiac rhythm disturbances or morphologic changes were detected except for in a few participants who appeared to have changes in their ECGs; the significance of the changes was unclear.	Not reported
Dawes et al,^31^ 2008, US	X26, 15 s	Law enforcement officers (21)	Nonrandomized trial (with a control group)	Low	CEW exposure was not associated with changes in core body temperature in resting, nonenvironmentally stressed adults.	Not reported
Ho et al,^32^ 2008, US	X26, 10 s	Law enforcement officers (34)	Nonrandomized trial (no control group)	Moderate	CEW exposure was not associated with clinically relevant tachyarrhythmias.	Not reported
Sloane et al,^33^ 2008, US	X26, 4.4 s (on average)	Law enforcement officers (66)	Nonrandomized trial (no control group)	Moderate	No abnormal serum troponin I levels were observed 6 h after exposure, suggesting no associated cardiac injuries.	Biosite Inc
Bozeman et al,^34^ 2009, US	X26, 5, 3, and 1 s	Law enforcement officers (28)	Nonrandomized trial (no control group)	Moderate	CEW exposure was not associated with detectable dysrhythmias; small increases in heart rate and systolic blood pressure were observed.	Department of Emergency Medicine
VanMeenen et al,^35^ 2013, US	X26, 5 s	Law enforcement trainees (23)	Nonrandomized trial (no control group)	Moderate	Cessation of normal breathing patterns (decrease in inspiratory activity) was observed during exposure. Normal breathing resumed after cessation of the exposure. There was no evidence of cardiac rhythm disruption.	National Institute of Justice
White et al,^36^ 2014, US	X26, duration not reported	Police officers (21)	Nonrandomized trial (no control group)	Moderate	CEW exposure was associated with significant reductions in cognitive functioning.	National Institute of Justice
Havranek et al,^37^ 2015, Czech Republic	X26, 5 s	Volunteers, type unclear (26)	Nonrandomized trial (no control group)	Moderate	CEW exposure was not associated with any clinically relevant ECG changes except for significant increase in heart rate in most participants.	Charles University, Prague
Gibbons et al,^38^ 2017, US	X26, 5 s	US Air Force personnel (24)	Nonrandomized trial (no control group)	High	CEW exposure was followed by significant changes in ECGs, suggesting a risk of ventricular tachycardia.	US Air Force
Ho et al,^39^ 2006, US	X26, 5 s	Taser CEW trainees (66)	Nonrandomized trial (no control group)	High	In a resting state, CEW exposure was not associated with significant changes in physiologic measures that could indicate dysrhythmias, cellular damage, hyperkalemia, or acidosis.	Axon Enterprise Inc
Ho et al,^40^ 2007, US	X26, 15 s or 3 times, each for 5 s	Law enforcement officers (52)	Nonrandomized trial (no control group)	Moderate	Prolonged (continuous or intermittent) CEW exposure was not associated with significant changes in respiratory parameters.	Axon Enterprise Inc
Dawes et al,^41^ 2009, US	X26, 5 s	Police officers (16)	Randomized clinical trial	Moderate	A 5-s CEW exposure did not induce a stronger physiologic stress response vs a 5-s exposure to oleoresin capsicum, a 45-s exposure of the hand and forearm in a 0 °C cold water tank, or a 1-min defensive tactics drill.	Axon Enterprise Inc
Ho et al,^42^ 2009, US	X26, 15 s	Taser CEW trainees (40)	Randomized clinical trial	Moderate	The group that was only exposed to CEWs showed higher pH and lower lactate values than those who completed the exertion protocol only. There were no significant differences between the groups in combination CEW exposure and exertion and exertion followed by continued exertion.	Axon Enterprise Inc
Dawes et al,^43^ 2010, US	X26, 5 s	Law enforcement officers (10)	Nonrandomized trial (no control group)	Moderate	CEW exposure did not electrically capture the human heart muscle when used with probe deployment.	Axon Enterprise Inc
Dawes et al,^44^ 2010, US	C2*, 30 s	Law enforcement officers (11)	Nonrandomized trial (no control group)	Moderate	CEW exposure was associated with mild lactic acidosis; no other important physiologic effects were found.	Axon Enterprise Inc
Dawes et al,^45^ 2010, US	Taser shockwave, 5 s (2-3 times)	Law enforcement officers and taser CEW trainees (16)	Nonrandomized trial (no control group)	Moderate	CEW exposure was associated with mild increases in creatine kinase and lactate levels; a nonsignificant trend toward reduced respiratory volume was observed. ECGs did not show any effects.	Axon Enterprise Inc
Moscati et al,^46^ 2010, US	X26, 15 s	Law enforcement officers and medical personnel (22)	Nonrandomized trial (no control group)	Moderate	Prolonged, continuous CEW exposure of resting adults with acute alcohol intoxication was not associated with clinically significant changes in markers of metabolic acidosis.	Axon Enterprise Inc
Dawes et al,^47^ 2011, US	X3, 10 s	Law enforcement officers and prison officers (56)	Nonrandomized trial (no control group)	Low	A higher number of probes deployed during exposure was associated with higher levels of creatine kinase. There were no significant changes in other vital signs.	Axon Enterprise Inc
Ho et al,^48^ 2011, US	NCGEW, 10 s	Law enforcement officers, prison officers, and taser CEW trainees (45)	Nonrandomized trial (no control group)	Moderate	Exposure to NGCEW1 was associated with cardiac capture in one participant and the product was not approved for public use. Exposure to a modified version (NGCEW2) was not associated with any significant health effects.	Axon Enterprise Inc
Ho et al,^49^ 2013, US	X26, 5 s	Law enforcement officers and prison officers (8)	Randomized clinical trial	Moderate	Effects on markers of acidosis and stress following CEW exposure were comparable (equal or smaller) to an 18 m sprint.	Axon Enterprise Inc
Dawes et al,^50^ 2014, US	X26, 5 s	Law enforcement officers and prison officers (13)	Nonrandomized trial (with a control group)	Moderate	CEW exposure was associated with a decline in neurocognitive functioning. These effects were transient, of questionable clinical significance, and returned to baseline after 1 h.	Axon Enterprise Inc
Ho et al,^51^ 2014, US	X2, 10 s	Law enforcement officers and prison officers (10)	Nonrandomized trial (no control group)	Moderate	No associations between CEW exposure and changes in physiologic measurements were observed, based on various measured parameters (cardiac, respiratory, venous pH, electrolytes, and creatine kinase).	Axon Enterprise Inc

Abbreviations: CEW, conducted electrical weapon; ECG, electrocardiogram.

Assessment of the 22 studies with a higher risk of bias also did not yield consistent associations (Table 2). Thirteen of these studies were financed by Axon Enterprise Inc. Exposure duration was, on average, somewhat higher compared with that in the studies with a lower bias risk. In one study,^44^ exposure reached 30 seconds, and the investigators reported that prolonged exposure to CEWs was associated with mild lactic acidosis. Some other findings suggested small increases in blood pressure and heart rate with CEW exposure, as well as in creatine kinase levels, but no significant changes in troponin levels, body temperature, respiratory rate, and metabolic effects were observed, even after alcohol consumption. When exposure to CEWs was compared with exposure to other types of police intervention (eg, pepper spray and a police dog search and attack exercise), neurocognitive ability was reduced for a maximum duration of 1 hour for all exposed groups, but group measures did not differ statistically significantly from each other.

Most industry-funded studies, as well as those funded by other sources, showed no statistically significant findings. Among the 17 studies that reported receiving funding from the manufacturer, 5 reported changes in the examined physiologic measurements that were generally described as mild, and 1 study reported more serious effects in 1 participant after exposure to an experimental device that was later not approved for use. Regarding the studies reporting another funding source, 6 mentioned statistically significant changes in some of the physiologic response levels, with 4 studies stating that the measures shortly returned to baseline. Three additional studies reported some statistically significant changes as well, but those were considered as not being clinically relevant or significant.

## Discussion

This systematic review identified and assessed evidence in the peer-reviewed literature during the past 20 years of the possible human health risks to individuals exposed to a taser CEW in the law enforcement context. With the search strategies used, our established a priori–defined inclusion criteria, and consideration of the methodologic soundness of the included studies, we identified 33 eligible studies. All studies were of experimental design and focused on outcomes such as physiologic stress responses, heart rate, blood pressure, arrhythmias, blood acidity, or cognitive performance.

Most of the studies suggested either no significant association between CEW exposure and adverse health outcomes or reported contradictory findings. Exposure to the electrical current of a CEW does not seem to be associated with serious health problems, especially when maximum duration ranges between 5 and 15 seconds. The risk for adverse health outcomes associated with CEW exposure can therefore be estimated as low. However, most studies on the topic had methodologic limitations; 22 of 33 studies had a higher risk of methodologic bias, particularly regarding sample size and selection. Nevertheless, both studies with a lower and a higher risk of bias showed in general no significant associations between exposure and the examined measures. In this review, an additional distinction was made based on the funding source of each study. From our assessment it was not apparent that the results of the Axon Enterprise Inc–financed studies were substantially more likely to suggest that CEW exposure is less harmful, compared with studies that were financed by other sources, as suggested by Azadani et al.^52^ However, the latter study had a different scope and approach and focused on the broader literature on the effects of electrical weapons, including animal studies. Nevertheless, a consistent difference we observed was that the Axon Enterprise Inc–sponsored studies seemed to analyze small sample sizes more frequently.

Representativeness of the study population and generalizability of the existing evidence constitute major limitations of the current body of literature on the topic. Most studies exclusively relied on law enforcement officers as the study population, meaning that recruited participants were generally healthy and physically fit and therefore not representative of the population that would usually encounter a CEW deployment. In several cases, a reward was promised (eg, a CEW for personal use) as an incentive to participate. It is therefore questionable whether the existing evidence constitutes a good representation of real-life field situations, since it is not possible to draw solid conclusions regarding exposure effects in potentially vulnerable populations or high-risk groups, such as pregnant women, people with psychiatric problems, or those under the influence of substances. The only studies introducing potential vulnerability factors, such as physical exhaustion and alcohol consumption, suggested no clinically significant associations.^42,46,48^ Taking the current findings into account, the health warning issued by Axon Enterprise Inc^9^ regarding vulnerable groups cannot be substantiated on the basis of existing human studies. However, that lack of evidence does not mean that the possibility of adverse health outcomes should be ignored, given that research evidence on the dose-response effects among potential high risk groups is, to our knowledge, nonexistent and not feasible owing to ethical restrictions.

Lack of generalizability seems to be a major shortcoming in even more aspects. For instance, in European countries, such as the Netherlands, the taser X2 is being used, however, in most of the reviewed studies, only the possible effects of the taser X26 were examined.^53^ This limited variation in the models reviewed also makes it difficult to differentiate health responses by taser CEW model or maximum exposure duration thresholds on the basis of the included studies. Furthermore, almost all of the reviewed studies were conducted in the US, a country with legal, cultural, and systemic differences compared with Western Europe, and it is unclear whether these findings are applicable to the situation in other countries.

Heterogeneity in examined health outcomes, definitions, and methodologic aspects makes it difficult to directly compare some of the results of the relevant literature. For example, some studies consider the wounds caused by the barbs of the CEW as injury, while other studies do not. In addition, all reviewed studies focused on physiologic measures/stressors (eg, levels of lactate, pH, blood pressure, and creatine kinase levels), despite the unclear clinical relevance and lack of a broadly accepted hypothesis behind the choice to investigate these outcomes, but research on symptoms (eg, headache, dizziness, and sleep problems) and pain reaction is scarce. Investigation of such stressors could probably provide some information regarding the occurrence of an extreme state, often referred to as excited delirium,^54^ which does not have a formal diagnosis.^55^

Amnesty International as well as Reuters news agency^6,7^ have pointed out the possible dangers associated with CEWs for vulnerable groups. Reuters has created a database with possible deaths due to CEW use mostly in the US based on media reports, self-report by the operator of the CEW, and, in a minority of cases, an autopsy report.^7^ In 163 of these cases, the autopsy report confirmed that CEW was the cause of death or one of the contributing factors.^56^ In many cases death occurred in people who were under the influence of drugs.^56^ In the literature, exposure to CEWs has rarely been documented as the sole cause of death; in several articles, a CEW was reported as a possible contributing factor in a number of mortality cases, when long-term and/or repetitive exposure was combined with drug use or cardiovascular disease (eAppendix 5 in the Supplement). Based on the current literature, it is not possible to state that there is a direct negative effect of CEW exposure on the health of vulnerable groups; at the same time, it has not been scientifically substantiated that use of a CEW on these groups is safe. The responsibility for the safe use of a CEW lies with the police officer who uses it and is well trained in all aspects of its use. To consider the extensive use of CEWs in law enforcement, it is crucial to look at both the advantages and disadvantages compared with the use of other weapons or police equipment. The use of firearms can often become lethal, while a CEW may possibly serve as a less-lethal substitute, minimizing physical violence while allowing a relatively secure distance from the possible perpetrator or threat. The use of batons and pepper spray is another alternative; however, there is possible risk associated with their use.^57,58^

There is in general little evidence on the risk of injuries associated with the stun mode. Studies tend to use alligator clips on the clothes during provocation tests instead of barbed darts shot into the skin or deeper into the body, given that purposefully putting the participants at risk of physical injury is not applicable and is not ethically acceptable. It is also difficult to estimate the actual risk of this type of injury in real-life conditions. Based on descriptive studies (eAppendix 2 in the Supplement), an estimate can be made of the risk of injury. According to El-Sayed et al,^59^ in approximately 1 of 200 000 cases, the CEW darts had to be removed in the hospital. Bozeman et al^60^ reported that, in 99.75% of CEW deployment cases, there were no injuries or only superficial injuries due to penetration from the darts. In other studies, however, the prevalence seems to be higher: according to Haileyesus et al,^61^ 11 of 100 cases involved nonfatal injuries treated in a hospital without any significant injuries among minors. In another study, 20% of a sample of minors had mild injuries, such as bruises and scrapes.^62^ A large multicenter study showed that the position of the exposed individual (prone vs nonprone) during exposure did not seem to be associated with sudden in-custody death rates.^63^ It is often challenging to compare these findings, given the differences in methods and the definition and documentation of the injuries. There is, in any case, some risk of injury with exposure to a CEW, but that chance appears to be small. eTable 3 in the Supplement provides a summary of case studies identified during the literature searches, categorizing the injuries per organ system.

Further investigation into the possible causal mechanisms between CEW use and human health effects is needed. Although a large part of the literature concerns animal studies, animal models do not necessarily approximate the possible effects of CEWs on the human body and the hypothesis that the impact of CEW exposure on animals (eg, swine) and humans is similar, seems to be problematic.^64,65,66^

For research transparency and replicability, the methods of experimental studies and clinical trials on the human health effects of CEWs should be preregistered in the form of an open-access protocol. Investigation of health outcomes could be expanded, assessing a broader range of possible health outcomes, such as somatic and psychological symptoms in association with CEW exposure. Although in terms of design, randomized clinical trials are used for the clarification of causal relationships, the methodologic flaws of the existing literature and ethical considerations in this field of research make the performance of observational studies the next timely step that would allow the investigation of longer-term outcomes at the population level. A reliable monitoring system should be established in which diagnosed health conditions, injuries, symptoms, and the extent to which a CEW was deployed will be routinely registered in primary, secondary, or forensic health care services.

### Strengths and Limitations

To our knowledge, this is the first systematic review that focused on the association between CEW exposure and human health effects based on a preregistered protocol and a comprehensive search strategy. No meta-analysis was possible owing to the high heterogeneity in the type and operationalization of the outcomes investigated in the reviewed studies.

## Conclusions

Based on currently published evidence in the peer-reviewed literature, the risk for adverse health outcomes associated with CEW exposure may be estimated as low, when the same deployment guidelines are applied as in the studies. However, it is not possible to draw conclusions about the extent to which the existing evidence constitutes a good representation of real-life field situations. For this reason, and until further data are available from more methodologically sound studies, it is recommended to follow the precautionary principle, when applicable. By systematically evaluating the health effects of CEW use in daily policing practice (physical as well as mental), it will become clear to what extent the results reported in this review apply to real-life conditions. The involvement of a physician or nurse in the health evaluation of cases in which a CEW was used and the development of a health care database in which the associated health outcomes would be routinely registered would provide further insight into the possible health impact of CEWs, especially for potential at-risk groups.

## Notes

Supplement. **eTable 1.** Types of Taser Weapons Documented in the Literature and Basic Characteristics **eTable 2.** Inclusion and Exclusion Criteria Form **eTable 3.** Summary of Case Studies on Documented Health Problems After CEW Exposure **eAppendix 1.** Modified Version of the AXIS Tool for the Assessment of Study Quality **eAppendix 2.** List of Descriptive Studies Identified During the Search **eAppendix 3.** List of Case-Studies Identified in the Literature Searches **eAppendix 4.** Reviews and Overviews Identified in the Literature Searches **eAppendix 5.** Studies on Reported Associations Between Death Incidents and Taser **eAppendix 6.** Database Searches

## References

[1] Robb M, Close B, Furyk J, Aitken P Emergency department implications of the TASER. Emerg Med Australas. 2009;21(4):250-258. 19682009 10.1111/j.1742-6723.2009.01194.x

[2] MacDonald JM, Kaminski RJ, Smith MR The effect of less-lethal weapons on injuries in police use-of-force events. Am J Public Health. 2009;99(12):2268-2274. doi:10.2105/AJPH.2009.159616 19846686 PMC2775771

[3] Taylor B, Woods DJ Injuries to officers and suspects in police use-of-force cases: a quasi-experimental evaluation. Police Quarterly. 2010;13(3):260-289. doi:10.1177/1098611110373994

[4] Soleimanirahbar A, Lee BK The TASER safety controversy. Expert Rev Med Devices. 2011;8(6):661-663. doi:10.1586/erd.11.53 22029461

[5] Dyer O. Tasers. *BMJ*. 2015;351:h6070. doi:10.1136/bmj.h6070 26577942

[6] Amnesty International. Use of taser by the Dutch police unacceptable. Published February 20, 2018 Accessed July 10, 2020. https://www.amnesty.nl/actueel/use-of-taser-by-the-dutch-police-unacceptable

[7] Cameron G. Reuters Investigates Shock tactics, part 5—the X26. Published September 21, 2017 Accessed August 27, 2020. https://www.reuters.com/investigates/special-report/usa-taser-x26/

[8] Laub J. Study of Deaths Following Electro Muscular Disruption. National Institute of Justice; 2011.

[9] Axon Enterprise Inc. TASER handheld CEW warnings, instructions, and information: law enforcement—important safety and health information. Published October 30, 2018 Accessed May 11, 2020. https://axon.cdn.prismic.io/axon%2F3cd3d65a-7500-4667-a9a8-0549fc3226c7_law-enforcement-warnings%2B8-5x11.pdf

[10] Moher D, Liberati A, Tetzlaff J, Altman DG; PRISMA Group Preferred Reporting Items for Systematic Reviews and Meta-analyses: the PRISMA statement. PLoS Med. 2009;6(7):e1000097. doi:10.1371/journal.pmed.1000097 19621072 PMC2707599

[11] Baliatsas C, Gerbecks J, Yzermans CJ, Dückers MLA Effects of exposure to conducted electrical weapons (tasers) on human health: a systematic review. Published September 9, 2019 Accessed July 10, 2020. https://www.crd.york.ac.uk/prospero/display_record.php?ID=CRD42019149472

[12] Higgins JP, Altman DG, Gøtzsche PC, ; Cochrane Bias Methods Group; Cochrane Statistical Methods Group The Cochrane Collaboration’s tool for assessing risk of bias in randomised trials. BMJ. 2011;343:d5928. doi:10.1136/bmj.d5928 22008217 PMC3196245

[13] Downes MJ, Brennan ML, Williams HC, Dean RS Development of a critical appraisal tool to assess the quality of cross-sectional studies (AXIS). BMJ Open. 2016;6(12):e011458. doi:10.1136/bmjopen-2016-011458 27932337 PMC5168618

[14] Sacolo H, Chimbari M, Kalinda C Knowledge, attitudes and practices on schistosomiasis in sub-Saharan Africa: a systematic review. BMC Infect Dis. 2018;18(1):46. doi:10.1186/s12879-017-2923-6 29347919 PMC5773048

[15] Downs SH, Black N The feasibility of creating a checklist for the assessment of the methodological quality both of randomised and non-randomised studies of health care interventions. J Epidemiol Community Health. 1998;52(6):377-384. doi:10.1136/jech.52.6.377 9764259 PMC1756728

[16] Robinson JK, Dellavalle RP, Bigby M, Callen JP Systematic reviews: grading recommendations and evidence quality. Arch Dermatol. 2008;144(1):97-99. doi:10.1001/archdermatol.2007.28 18209174

[17] Landis JR, Koch GG The measurement of observer agreement for categorical data. Biometrics. 1977;33(1):159-174. doi:10.2307/2529310 843571

[18] Mandrekar JN Measures of interrater agreement. J Thorac Oncol. 2011;6(1):6-7. doi:10.1097/JTO.0b013e318200f983 21178713

[19] Vilke GM, Sloane CM, Bouton KD, Physiological effects of a conducted electrical weapon on human subjects. Ann Emerg Med. 2007;50(5):569-575. doi:10.1016/j.annemergmed.2007.05.004 17719689

[20] Vilke GM, Sloane C, Levine S, Neuman T, Castillo E, Chan TC Twelve-lead electrocardiogram monitoring of subjects before and after voluntary exposure to the taser X26. Am J Emerg Med. 2008;26(1):1-4. doi:10.1016/j.ajem.2007.01.005 18082773

[21] Vilke GM, Sloane CM, Suffecool A, Physiologic effects of the taser after exercise. Acad Emerg Med. 2009;16(8):704-710. doi:10.1111/j.1553-2712.2009.00458.x 19594461

[22] VanMeenen KM, Cherniack NS, Bergen MT, Gleason LA, Teichman R, Servatius RJ Cardiovascular evaluation of electronic control device exposure in law enforcement trainees: a multisite study. J Occup Environ Med. 2010;52(2):197-201. doi:10.1097/JOM.0b013e3181cc58ba 20134349

[23] Kane RJ, White MD Taser exposure and cognitive impairment implications for valid Miranda waivers and the timing of police custodial interrogations. Criminol Public Policy. 2016;15(1):79-107. doi:10.1111/1745-9133.12173

[24] Stopyra JP, Winslow JE, Fitzgerald DM, Bozeman WP Intracardiac electrocardiographic assessment of precordial taser shocks in human subjects: a pilot study. J Forensic Leg Med. 2017;52:70-74. doi:10.1016/j.jflm.2017.08.004 28866284

[25] Kroll MW, Hail SL, Kroll RM, Wetli CV, Criscione JC Electrical weapons and excited delirium: shocks, stress, and serum serotonin. Forensic Sci Med Pathol. 2018;14(4):478-483. doi:10.1007/s12024-018-0005-8 30099702

[26] Ho JD, Dawes DM, Bultman LL, Moscati RM, Janchar TA, Miner JR Prolonged taser use on exhausted humans does not worsen markers of acidosis. Am J Emerg Med. 2009;27(4):413-418. doi:10.1016/j.ajem.2008.03.017 19555610

[27] Ho JD, Dawes DM, Nelson RS, Acidosis and catecholamine evaluation following simulated law enforcement “use of force” encounters. Acad Emerg Med. 2010;17(7):e60-e68. doi:10.1111/j.1553-2712.2010.00813.x 20653572

[28] Ho JD, Dawes DM, Heegaard WG, Calkins HG, Moscati RM, Miner JR Absence of electrocardiographic change after prolonged application of a conducted electrical weapon in physically exhausted adults. J Emerg Med. 2011;41(5):466-472. doi:10.1016/j.jemermed.2009.03.023 19443165

[29] Dawes D, Ho J, Vincent AS, Nystrom P, Driver B The neurocognitive effects of a conducted electrical weapon compared to high intensity interval training and alcohol intoxication—implications for Miranda and consent. J Forensic Leg Med. 2018;53:51-57. doi:10.1016/j.jflm.2017.11.001 29172160

[30] Levine SD, Sloane CM, Chan TC, Dunford JV, Vilke GM Cardiac monitoring of human subjects exposed to the taser. J Emerg Med. 2007;33(2):113-117. doi:10.1016/j.jemermed.2007.02.018 17692758

[31] Dawes DM, Ho JD, Johnson MA, Lundin E, Janchar TA, Miner JR 15-Second conducted electrical weapon exposure does not cause core temperature elevation in non-environmentally stressed resting adults. Forensic Sci Int. 2008;176(2-3):253-257. doi:10.1016/j.forsciint.2007.09.014 17983716

[32] Ho JD, Dawes DM, Reardon RF, Echocardiographic evaluation of a Taser-X26 application in the ideal human cardiac axis. Acad Emerg Med. 2008;15(9):838-844. doi:10.1111/j.1553-2712.2008.00201.x 19244634

[33] Sloane CM, Chan TC, Levine SD, Dunford JV, Neuman T, Vilke GM Serum troponin I measurement of subjects exposed to the Taser X-26. J Emerg Med. 2008;35(1):29-32. doi:10.1016/j.jemermed.2007.08.073 18296010

[34] Bozeman WP, Barnes DG Jr, Winslow JE III, Johnson JC III, Phillips CH, Alson R Immediate cardiovascular effects of the Taser X26 conducted electrical weapon. Emerg Med J. 2009;26(8):567-570. doi:10.1136/emj.2008.063560 19625551

[35] VanMeenen KM, Lavietes MH, Cherniack NS, Bergen MT, Teichman R, Servatius RJ Respiratory and cardiovascular response during electronic control device exposure in law enforcement trainees. Front Physiol. 2013;4:78. doi:10.3389/fphys.2013.00078 23616772 PMC3629983

[36] White MD, Ready JT, Kane RJ, Dario LM Examining the effects of the TASER on cognitive functioning: findings from a pilot study with police recruits. J Exp Criminol. 2014;10(3):267-290. doi:10.1007/s11292-013-9197-9

[37] Havranek S, Neuzil P, Linhart A Electromuscular incapacitating devices discharge and risk of severe bradycardia. Am J Forensic Med Pathol. 2015;36(2):94-98. doi:10.1097/PAF.0000000000000143 25710795 PMC4927311

[38] Gibbons JA, Mojica AJ, Peele ME Human electrical muscular incapacitation and effects on QTc interval. J Forensic Sci. 2017;62(6):1516-1521. doi:10.1111/1556-4029.13490 28419455

[39] Ho JD, Miner JR, Lakireddy DR, Bultman LL, Heegaard WG Cardiovascular and physiologic effects of conducted electrical weapon discharge in resting adults. Acad Emerg Med. 2006;13(6):589-595. doi:10.1197/j.aem.2006.01.017 16551780

[40] Ho JD, Dawes DM, Bultman LL, Respiratory effect of prolonged electrical weapon application on human volunteers. Acad Emerg Med. 2007;14(3):197-201. doi:10.1197/j.aem.2006.11.016 17284465

[41] Dawes D, Ho J, Miner J The neuroendocrine effects of the TASER X26: a brief report. Forensic Sci Int. 2009;183(1-3):14-19. doi:10.1016/j.forsciint.2008.09.015 19019594

[42] Ho JD, Dawes DM, Cole JB, Hottinger JC, Overton KG, Miner JR Lactate and pH evaluation in exhausted humans with prolonged TASER X26 exposure or continued exertion. Forensic Sci Int. 2009;190(1-3):80-86. doi:10.1016/j.forsciint.2009.05.016 19539437

[43] Dawes DM, Ho JD, Reardon RF, Miner JR Echocardiographic evaluation of TASER X26 probe deployment into the chests of human volunteers. Am J Emerg Med. 2010;28(1):49-55. doi:10.1016/j.ajem.2008.09.033 20006201

[44] Dawes DM, Ho JD, Reardon RF, Miner JR The cardiovascular, respiratory, and metabolic effects of a long duration electronic control device exposure in human volunteers. Forensic Sci Med Pathol. 2010;6(4):268-274. doi:10.1007/s12024-010-9166-9 20502988

[45] Dawes DM, Ho JD, Reardon RF, Sweeney JD, Miner JR The physiologic effects of multiple simultaneous electronic control device discharges. West J Emerg Med. 2010;11(1):49-56. 20411076 PMC2850854

[46] Moscati R, Ho JD, Dawes DM, Miner JR Physiologic effects of prolonged conducted electrical weapon discharge in ethanol-intoxicated adults. Am J Emerg Med. 2010;28(5):582-587. doi:10.1016/j.ajem.2009.02.010 20579553

[47] Dawes DM, Ho JD, Reardon RF, The respiratory, metabolic, and neuroendocrine effects of a new generation electronic control device. Forensic Sci Int. 2011;207(1-3):55-60. doi:10.1016/j.forsciint.2010.08.028 20884143

[48] Ho JD, Dawes DM, Reardon RF, Human cardiovascular effects of a new generation conducted electrical weapon. Forensic Sci Int. 2011;204(1-3):50-57. doi:10.1016/j.forsciint.2010.05.003 20537475

[49] Ho JD, Dawes DM, Nystrom PC, Markers of acidosis and stress in a sprint versus a conducted electrical weapon. Forensic Sci Int. 2013;233(1-3):84-89. doi:10.1016/j.forsciint.2013.08.022 24314505

[50] Dawes DM, Ho JD, Vincent AS, The neurocognitive effects of simulated use-of-force scenarios. Forensic Sci Med Pathol. 2014;10(1):9-17. doi:10.1007/s12024-013-9510-y 24213973

[51] Ho JD, Dawes DM, Chang RJ, Nelson RS, Miner JR Physiologic effects of a new-generation conducted electrical weapon on human volunteers. J Emerg Med. 2014;46(3):428-435. doi:10.1016/j.jemermed.2013.08.069 24238599

[52] Azadani PN, Tseng ZH, Ermakov S, Marcus GM, Lee BK Funding source and author affiliation in TASER research are strongly associated with a conclusion of device safety. Am Heart J. 2011;162(3):533-537. doi:10.1016/j.ahj.2011.05.025 21884872

[53] CAST assessment of the Taser X2 against the police operational requirements. Published January 26, 2018 Accessed July 10, 2020. https://www.gov.uk/government/publications/cast-assessment-of-the-taser-x2

[54] Vilke GM, DeBard ML, Chan TC, Excited delirium syndrome (ExDS): defining based on a review of the literature. J Emerg Med. 2012;43(5):897-905. doi:10.1016/j.jemermed.2011.02.017 21440403

[55] Gonin P, Beysard N, Yersin B, Carron PN Excited delirium: a systematic review. Acad Emerg Med. 2018;25(5):552-565. doi:10.1111/acem.13330 28990246

[56] Reuters Investigates Shock tactics: a Reuters examination of 1081 deaths involving Tasers. Accessed September 24, 2020. https://www.reuters.com/investigates/special-report/usa-taser-database/

[57] Payne-James JJ Restraint techniques, injuries, and death: baton In: Encyclopedia of Forensic and Legal Medicine. 2nd ed Elsevier; 2016:115-117. doi:10.1016/B978-0-12-800034-2.00326-8

[58] Kearney T, Hiatt P, Birdsall E, Smollin C Pepper spray injury severity: ten-year case experience of a poison control system. Prehosp Emerg Care. 2014;18(3):381-386. doi:10.3109/10903127.2014.891063 24669935

[59] El Sayed M, El Tawil C, Tamim H, Mailhac A, Mann NC Emergency medical services experience with barb removal after taser use by law enforcement: a descriptive national study. Prehosp Disaster Med. 2018;34:1-8. 30591087 10.1017/S1049023X18001176

[60] Bozeman WP, Hauda WE II, Heck JJ, Graham DD Jr, Martin BP, Winslow JE Safety and injury profile of conducted electrical weapons used by law enforcement officers against criminal suspects. Ann Emerg Med. 2009;53(4):480-489. doi:10.1016/j.annemergmed.2008.11.021 19157651

[61] Haileyesus T, Annest JL, Mercy JA Non-fatal conductive energy device-related injuries treated in US emergency departments, 2005-2008. Inj Prev. 2011;17(2):127-130. doi:10.1136/ip.2010.028704 21257680

[62] Gardner AR, Hauda WE II, Bozeman WP Conducted electrical weapon (TASER) use against minors: a shocking analysis. Pediatr Emerg Care. 2012;28(9):873-877. doi:10.1097/PEC.0b013e31826763d1 22929134

[63] Hall C, Votova K, Heyd C, Restraint in police use of force events: examining sudden in custody death for prone and not-prone positions. J Forensic Leg Med. 2015;31:29-35. doi:10.1016/j.jflm.2014.12.007 25735781

[64] Pound P, Ebrahim S, Sandercock P, Bracken MB, Roberts I; Reviewing Animal Trials Systematically (RATS) Group Where is the evidence that animal research benefits humans? BMJ. 2004;328(7438):514-517. doi:10.1136/bmj.328.7438.514 14988196 PMC351856

[65] Pippin JJ Taser research in pigs not helpful. J Am Coll Cardiol. 2007;49(6):731-732. doi:10.1016/j.jacc.2006.11.019 17291944

[66] Jauchem JR Deaths in custody: are some due to electronic control devices (including TASER devices) or excited delirium? J Forensic Leg Med. 2010;17(1):1-7. doi:10.1016/j.jflm.2008.05.011 20083043

