# Supplementary material for: Human Health Risks of Conducted Electrical Weapon Exposure: A Systematic Review
Source: JAMA Netw Open. 2021 Feb 12;4(2):e2037209. doi: 10.1001/jamanetworkopen.2020.37209 (PMC7881359; doi:10.1001/jamanetworkopen.2020.37209)
Supplement: Supplement. — eTable 1. Types of Taser Weapons Documented in the Literature and Basic Characteristics eTable 2. Inclusion and Exclusion Criteria Form eTable 3. Summary of Case Studies on Documented Health Problems After CEW Exposure eAppendix 1. Modified Version of the AXIS Tool for the Assessment of Study Quality eAppendix 2. List of Descriptive Studies Identified During the Search eAppendix 3. List of Case-Studies Identified in the Literature Searches eAppendix 4. Reviews and Overviews Identified in the Literature Searches eAppendix 5. Studies on Reported Associations Between Death Incidents and Taser eAppendix 6. Database Searches [file jamanetwopen-e2037209-s001.pdf]

Supplementary Online Content

Baliatsas C, Gerbecks J, Dückers MLA, Yzermans CJ. Human health risks of conducted electrical weapon exposure: a systematic review. *JAMA Netw Open*. 2021;4(2):e2037209. doi:10.1001/jamanetworkopen.2020.37209

eTable 1. Types of Taser Weapons Documented in the Literature and Basic Characteristics
eTable 2. Inclusion and Exclusion Criteria Form
eTable 3. Summary of Case Studies on Documented Health Problems After CEW Exposure
eAppendix 1. Modified Version of the AXIS Tool for the Assessment of Study Quality
eAppendix 2. List of Descriptive Studies Identified During the Search
eAppendix 3. List of Case-Studies Identified in the Literature Searches
eAppendix 4. Reviews and Overviews Identified in the Literature Searches
eAppendix 5. Studies on Reported Associations Between Death Incidents and Taser
eAppendix 6. Database Searches

This supplementary material has been provided by the authors to give readers additional information about their work.

© 2021 Baliatsas C et al. *JAMA Network Open.*

eTable 1. Types of Taser weapons documented in the literature and basic characteristics*

Type (production year)	Electrical charge**	Additional details
AIR TASER® (1993)	70 μC	
M26 (1999)	70 – 120 μC	
X26 (2003)	80 – 135 μC	Not available since 2014
X3 (2009)	63 (+/- 9) μC	It fires two barbed darts
X2 (2011)	63 (+/- 9) μC	It fires two barbed darts in one cartridge; the electric current lasts a maximum of 5 seconds for every activation
X26P (2013)	63 (+/- 9) μC	Compact version of X26 (in terms of produced charge, weight and size)
X7 (2018)	Not documented	Newest version; has the features of the X2, but also toggles between cartridges by flicking the weapon, simultaneously reloads two cartridges, and is close-range optimized

* There are more TASER® CEW types available for civilian use. In the present table only CEW types extensively used in law enforcement are presented.
** Measured in microcoulombs (μC).
Source: Reuters Investigates. Shock Tactics – Part 5 – the X26. 2017. Retrieved from https://www.reuters.com/investigates/special-report/usa-taser-x26/

© 2021 Baliatsas C et al. *JAMA Network Open.*

eTable 2. Inclusion and exclusion criteria form

Specifications regarding Inclusion/Exclusion criteria	
**Publication characteristics**	**Criteria**
Publication language	English, Dutch, French, German, Spanish.
Publication status	Primary scientific studies on original data, published in peer-reviewed journals (as a minimum, fundamental criterion of quality and validity). Studies only reported in books, reports without peer-review, conference proceedings/abstracts, dissertations, and protocols (without findings) are excluded. In the case of "double" publications/multiple articles based on the same data, the original/first publication is included as reference.
Publication period	01/2000 – 04/2019 (04/2020 after the update search)
**Study characteristics**	**Criteria**
Study design	No restriction, except for case studies (1 person per investigation as sample).
Study population	No restriction regarding human samples (studies on healthy individuals as well as patients with specific conditions are eligible for inclusion). Also no restriction in terms of demographic characteristics. Animal studies are excluded.
Health outcome	No restriction.
Health outcome assessment	Self-reported and/or diagnosed/objectively assessed health symptoms and conditions, health status/symptom scores and/or physiological measures, as dependent variables. Studies that did not examine specific health/physiological outcomes in relation to CEW exposure are not eligible for inclusion.
Presence of somatic and/or psychological (co-)morbidity	No restriction.
Sample age	No restriction.
Sample size	No restriction.
**Exposure**	**Criteria**
Type of exposure (source)	Studies focusing on TASER® CEWs (all models from Taser International/Axon), more specifically: M26, X26(P), X3, X2, XREP (and also more recent models), as primary exposure source. Studies on guns/devices that do not fall within this classification, such as "stun guns", "batons"/"prods" and "belts" are excluded.

© 2021 Baliatsas C et al. *JAMA Network Open.*

eTable 3. Summary of case studies on documented health problems after CEW exposure

Organ system	Type of conditions	N studies
**Eye**	Penetration of eye (region) by darts	37
**Cardiovascular**	Atrial fibrillation, ventricular fibrillation, cardiacarrest , chest pain, asystole, myocardial capture, tachycardia	26
**Skin**	Skin burns, stab wound	23*
**Neurological**	Sixteen times head trauma/injury (caused by a fall), in 8 cases this led to death, in 8 other cases it 'supposedly' led to death<br>Epilepsy	19**
**Skull**	Penetration of the skull by darts	10
**Muskoskeletal**	Fractures of different body parts, rhadbdomyolysis	10
**Respiratory**	Air between the lungs, pneumothorax	2
**Pharynx**	Perforation of the pharynx by dart	1
**Male genitals**	Penetration by dart	1
**Other**	Excited delirium	1

* of which 22 from one study
** of which 16 from one study

© 2021 Baliatsas C et al. *JAMA Network Open.*

eAppendix 1. Modified version of the AXIS tool for the assessment of study quality**

Introduction

1. Clear definition of objectives/ aims?

Methods

2. Study design appropriate for the stated aims?
3. Was the sample size justified?
4. Target population clearly defined (appropriate population base/unbiased sampling)?
5. Risk factor and outcome variables measured correctly using instruments that had been trialed, piloted or published previously?
6. Methods (including statistical methods) sufficiently described to enable them to be repeated?
7. Was an attempt made to blind those measuring the main outcomes of the intervention? (*Note: For all experimental studies*)*
8. Were study subjects randomised to intervention groups? *(Note: For controlled trials*)*

Results

9. Were the results for analysis described in the methods, adequately presented?
10. Were the authors' discussions and conclusions justified by the results?

Disussion

11. Were the limitations of the study discussed?
12 Was ethical approval or consent of participants attained?

** *The total quality score can vary between 0 and 10 where 0-4 = (Low); 5-7 = (Moderate) and 8-10= (High). For Controlled trials (CTs) two extra items were used (7, 8) and the total score is adjusted accordingly: 0-6 = (Low); 7-9 = (Moderate) and 10-12= (High). For experimental studies/interventions without a control group, one extra item is used (7) and the total score is adjusted accordingly: 0-5 = (Low); 6-8 = (Moderate) and 9-11= (High).*

**Added from from Downs and Black, 1998*

© 2021 Baliatsas C et al. *JAMA Network Open.*

eAppendix 2. List of descriptive studies identified during the search

Bécour B. Conducted electrical weapons: About 42 cases examined in emergency department. *Revue de Medecine Legale*. 2012; 3:57-63.

Bozeman WP, Hauda IWE, Heck JJ, Graham Jr DD, Martin BP, Winslow JE. Safety and Injury Profile of Conducted Electrical Weapons Used by Law Enforcement Officers Against Criminal Suspects. *Annals of emergency medicine*. 2009; 53:480-9.

Bozeman WP, Teacher E, Winslow JE. Transcardiac conducted electrical weapon (TASER) probe deployments: incidence and outcomes. *The Journal of emergency medicine*. 2012; 43:970-5.

Coombs AV, Eyerly-Webb SA, Solomon RJ, Sanchez R, Lee SK, Carrillo EH, et al. Investigating clinical and cost burdens of law enforcement–related K9 injuries: The impact of “the bite” on a community hospital. *American Surgeon*. 2019; 85:64-70.

DeLone GJ, Thompson LM. The application and use of TASERs by a Midwestern police agency. *International Journal of Police Science & Management*. 2009;11:414-28.

Eastman AL, Metzger JC, Pepe PE, Benitez FL, Decker J, Rinnert KJ, et al. Conductive electrical devices: a prospective, population-based study of the medical safety of law enforcement use. *The Journal of trauma*. 2008; 64:1567-72.

El Sayed M, El Tawil C, Tamim H, Mailhac A, Mann NC. Emergency Medical Services Experience With Barb Removal After Taser Use By Law Enforcement: A Descriptive National Study. *Prehospital and Disaster Medicine*. 2019; 34:38-45.

Feldman JM, Gruskin S, Coull BA, Krieger N. Quantifying underreporting of law-enforcement-related deaths in United States vital statistics and news-media-based data sources: A capture–recapture analysis. *PLoS medicine*. 2017; 14.

Gardner AR, Hauda WE, Bozeman WP. Conducted electrical weapon (TASER) use against minors: A shocking analysis. *Pediatric emergency care*. 2012; 28:873-7.

Haileyesus T, Annest JL, Mercy JA. Non-fatal conductive energy device-related injuries treated in US emergency departments, 2005-2008. *Injury Prevention*. 2011; 17:127-30.

Jenkinson E, Neeson C, Bleetman A. The relative risk of police use-of-force options: Evaluating the potential for deployment of electronic weaponry. *Journal of clinical forensic medicine*. 2006; 13:229-41.

Lee BK, Vittinghoff E, Whiteman D, Park M, Lau LL, Tseng ZH. Relation of Taser (Electrical Stun Gun) Deployment to Increase in In-Custody Sudden Deaths. *American Journal of Cardiology*. 2009; 103:877-80.

Swerdlow CD, Fishbein MC, Chaman L, Lakkireddy DR, Tchou P. Presenting rhythm in sudden deaths temporally proximate to discharge of TASER conducted electrical weapons. *Academic emergency medicine*. 2009; 16:726-39.

Strote J, Walsh M, Angelidis M, Basta A, Hutson HR. Conducted electrical weapon use by law enforcement: An evaluation of safety and injury. *Journal of Trauma - Injury, Infection and Critical Care*. 2010; 68:1239-46.

White MD, Ready J. Examining fatal and nonfatal incidents involving the TASER: Identifying predictors of suspect death reported in the media. *Criminology & Public Policy*. 2009;8:865-91.

White MD, Ready J, Riggs C, Dawes DM, Hinz A, Ho JD. An incident-level profile of TASER device deployments in arrest-related deaths. *Police Quarterly*. 2013; 16:85-112.

© 2021 Baliatsas C et al. *JAMA Network Open.*

eAppendix 3. List of case-studies identified in the literature searches

Al-Jarabah M, Coulston J, Hewin D. Pharyngeal perforation secondary to electrical shock from a Taser gun. *Emergency medicine journal*. 2008; 25:378.

Baldwin DE, Nagarakanti R, Hardy SP, Jain N, Borne DM, England AR, et al. Myocardial infarction after taser exposure. *The Journal of the Louisiana State Medical Society*. 2010; 162:291-2, 4-5.

Belen E, Tipi FF, Bayyigit A, Helvaci AS. Acute inferior myocardial infarction after electrical weapon exposure: case report and review of the literature. *Turk Kardiyoloji Dernegi arsivi*. 2015; 43:178-81.

Bell N, Moon M, Dross P. Cerebrovascular accident (CVA) in association with a Taser-induced electrical injury. *Emergency radiology*. 2014; 21:211-3.

Ben Ahmed H, Bouzouita K, Selmi K, Chelli M, Mokaddem A, Ben Ameur Y, et al. Myocardial infarction after conducted electrical weapon shock. *Annales de cardiologie et d'angeiologie*. 2013; 62:124-6.

Le Blanc-Louvry I, Gricourt C, Toure E, Papin F, Proust B. A brain penetration after Taser injury: controversies regarding Taser gun safety. *Forensic science international*. 2012; 221:e7-11.

Bui ET, Sourkes M, Wennberg R. Generalized tonic-clonic seizure after a taser shot to the head. *Canadian Medical Association Journal*. 2009; 180:625-6.

Bux R, Andresen D, Rothschild MA. Electronic gun Advanced Taser M 26: Functioning, effectiveness and case report. *Rechtsmedizin*. 2002; 12:207-13.

Campbell F, Clark S. Penetrating facial trauma from a Taser barb. *British Journal of Oral & Maxillofacial Surgery*. 2019; 57:188-9.

Cao M, Shinbane JS, Gillberg JM, Saxon LA. Taser-induced rapid ventricular myocardial capture demonstrated by pacemaker intracardiac electrograms. *Journal of cardiovascular electrophysiology*. 2007; 18:876-9.

Chandler J, Martin BP, Graham DD, Jr. TASER((R)) injury to the forehead. *The Journal of emergency medicine*. 2013; 44:e67-8.

Cheek S, Shifflette V, Dunn E. Foreign body removal: A shocking story. *American Surgeon*. 2013; 79:E35-E6.

Chen SL, Richard CK, Murthy RC, Lauer AK. Perforating ocular injury by Taser. *Clinical & experimental ophthalmology*. 2006; 34:378-80.

Coad F, Maw G. TASERed during training: an unusual scapular fracture. *Emergency medicine Australasia*. 2014; 26:206-7.

Cooks T, Davis TK, Hu J, Metheny R, Schwartz M, Gerona R. "Smoking" guns: Questions. "Smoking" guns: Answers. *Pediatric nephrology*. 2016; 31:61-2, 3-6.

De Runz A, Minetti C, Brix M, Simon E. New TASER injuries: Lacrimal canaliculus laceration and ethmoid bone fracture. *International journal of oral and maxillofacial surgery*. 2014; 43:722-4.

Dearing M, Lewis TJ. Foreign body lodged in distal phalanx of left index finger-taser dart. *Emergency radiology*. 2005; 11:364-5.

Dunet B, Erbland A, Abi-Chahla ML, Tournier C, Fabre T. The TASERed finger: A new entity. Case report and review of literature. *Chirurgie de la main*. 2015; 34:145-8.

Feeney C, Vu J, Ani C. Acute agitated delirious state associated with Taser exposure. *Journal of the National Medical Association*. 2010; 102:1254-7.

Gapsis BC, Hoang A, Nazari K, Morcos M. Ocular manifestations of TASER-induced trauma. *Trauma Case Reports*. 2017; 12:4-7.

Giaconi JC, Ries MD, Steinbach LS. Stun gun induced myotendinous injury of the iliopsoas and gluteus minimus. *Skeletal radiology*. 2011; 40:783-7.

Gleason J, Ahmad I. TASER® electronic control device-induced rhabdomyolysis and renal failure: A case report. *Journal of Clinical and Diagnostic Research*. 2015; 9:HD01-HD2.

Haegeli LM, Sterns LD, Adam DC, Leather RA. Effect of a Taser shot to the chest of a patient with an implantable defibrillator. *Heart rhythm*. 2006; 3:339-41.

Han JS, Chopra A, Carr D. Ophthalmic injuries from a TASER. *Cjem*. 2009; 11:90-3.

© 2021 Baliatsas C et al. *JAMA Network Open.*

Hinchey PR, Subramaniam G. Pneumothorax as a complication after TASER activation. *Prehospital emergency care*. 2009; 13:532-5.

Ho J, Lapine A, Joing S, Reardon R, Dawes D. Confirmation of respiration during trapezial conducted electrical weapon application. *Academic emergency medicine*. 2008; 15:398.

Jey A, Hull P, Kravchuk V, Carillo B, Martel JB. Emergent diagnosis and management of TASER penetrating ocular injury. *American Journal of Emergency Medicine*. 2016; 34:1740.e3-.e5.

Kaloostian PE, Tran H. Intracranial taser dart penetration: Literature review and surgical management. *Journal of surgical case reports*. 2012; 1:10.

Kroll MW, Lakkireddy DR, Stone JR, Luceri RM. TASER electronic control devices and cardiac arrests: coincidental or causal? *Circulation*. 2014; 129:93-100.

Kroll MW, Ritter MB, Guilbault RA, Panescu D. Infection Risk From Conducted Electrical Weapon Probes: What Do We Know? *Journal of forensic sciences*. 2016; 61:1556-62.

Kroll MW, Adamec J, Wetli CV, Williams HE. Fatal traumatic brain injury with electrical weapon falls. *Journal of Forensic and Legal Medicine*. 2016; 43:12-9.

Kroll MW, Ritter MB, Kennedy EA, Silverman NK, Shinder R, Brave MA, et al. Eye injuries from electrical weapon probes: Incidents, prevalence, and legal implications. *Journal of forensic and legal medicine*. 2018; 55:52-7.

Lewis MC, Lewis DE. Frontal Sinus TASER Dart Injury. *The Journal of emergency medicine*. 2016; 50:490-2.

Li JY, Hamill MB. Catastrophic globe disruption as a result of a TASER injury. *Journal of Emergency Medicine*. 2013; 44:65-7.

Little J, Burt M. Tasers and psychiatry: The use of a Taser on a low secure unit. *Journal of Psychiatric Intensive Care*. 2013; 9:56-8.

Maher PJ, Beck N, Strote J. Pneumomediastinum and pulmonary interstitial emphysema after tracheal taser injury. *Emergency medicine journal* : EMJ. 2015; 32:90.

Mangus BE, Shen LY, Helmer SD, Maher J, Smith RS. Taser and taser associated injuries: A case series. *American Surgeon*. 2008; 74:862-5.

Moysidis SN, Koulisis N, Rodger DC, Chao JR, Leng T, de Carlo T, et al. Thomas A. Swift's Electric Rifle Injuries to the Eye and Ocular Adnexa: The Management of Complex Trauma. *Ophthalmology Retina*. 2019; 3:258-69.

Multerer S, Berkenbosch JW, Das B, Johnsrude C. Atrial fibrillation after taser exposure in a previously healthy adolescent. *Pediatric emergency care*. 2009; 25:851-3.

Naunheim RS, Treaster M, Aubin C. Ventricular fibrillation in a man shot with a Taser. *Emergency medicine journal*. 2010; 27:645-6.

Ng W, Chehade M. Taser penetrating ocular injury. *American journal of ophthalmology*. 2005;139:713-5.

Rafailov L, Temnogorod J, Tsai FF, Shinder R. Impaled Orbital TASER Probe Injury Requiring Primary Enucleation. *Ophthalmic plastic and reconstructive surgery*. 2017; 33:S176-s7.

Rehman TU, Yonas H, Marinaro J. Intracranial penetration of a TASER dart. *The American journal of emergency medicine*. 2007; 25:733.e3-4.

Richards KA, Kleuser LP, Kluger J. Fortuitous therapeutic effect of Taser shock for a patient in atrial fibrillation. *Annals of emergency medicine*. 2008; 52:686-8.

Sanford JM, Jacobs GJ, Roe EJ, Terndrup TE. Two patients subdued with a TASER(R) device: cases and review of complications. *The Journal of emergency medicine*. 2011; 40:28-32.

Sayegh RR, Madsen KA, Adler JD, Johnson MA, Mathews MK. Diffuse retinal injury from a non-penetrating TASER dart. *Documenta Ophthalmologica*. 2011; 123:135-9.

Schwarz ES, Barra M, Liao MM. Successful resuscitation of a patient in asystole after a TASER injury using a hypothermia protocol. *American Journal of Emergency Medicine*. 2009; 27:515.e1-.e2.

Seth RK, Abedi G, Daccache AJ, Tsai JC. Cataract secondary to electrical shock from a Taser gun. *Journal of cataract and refractive surgery*. 2007; 33:1664-5.

Sharma A, Theivacumar NS, Souka HM. Tasers--less than lethal! *Annals of the Royal College of Surgeons of England*. 2009; 91:W20-1.

Sloane CM, Chan TC, Vilke GM. Thoracic Spine Compression Fracture After TASER Activation. *Journal of Emergency Medicine*. 2008; 34:283-5.

Strote J, Range Hutson H. Taser use in restraint-related deaths. *Prehospital emergency care*. 2006; 10:447-50.

Swerdlow CD, Fishbein MC, Chaman L, Lakkireddy DR, Tchou P. Presenting rhythm in sudden deaths temporally proximate to discharge of TASER conducted electrical weapons. *Academic emergency medicine*. 2009; 16:726-39.

Teymoorian S, San Filippo AN, Poulose AK, Lyon DB. Perforating globe injury from Taser trauma. *Ophthalmic plastic and reconstructive surgery*. 2010; 26:306-8.

Theisen K, Slater R, Hale N. Taser-Related Testicular Trauma. *Urology*. 2016; 88:e5.

Todak NE, Cesar GT, Louton B. Forensic reporting of TASER exposure: An examination of situational and exposure characteristics. *Journal of forensic and legal medicine*. 2015; 35:4-8.

Tyagi AC, Gill A, Felton B. Thoracic Compression Fracture as a Result of Taser® Discharge. *Clinical practice and cases in emergency medicine*. 2017;:319.

Vanga SR, Bommana S, Kroll MW, Swerdlow C, Lakkireddy D. TASER conducted electrical weapons and implanted pacemakers and defibrillators. *Engineering in Medicine and Biology Society IEEE*. 2009:3199-204.

Winslow JE, Bozeman WP, Fortner MC, Alson RL. Thoracic compression fractures as a result of shock from a conducted energy weapon: a case report. *Annals of emergency medicine*. 2007; 50:584-6.

Zipes DP. Sudden cardiac arrest and death following application of shocks from a TASER electronic control device. *Circulation*. 2012; 125:2417-22.

Zipes DP. TASER electronic control devices can cause cardiac arrest in humans. *Circulation*. 2014; 129:101-11.

© 2021 Baliatsas C et al. *JAMA Network Open.*

eAppendix 4. Reviews and overviews identified in the literature searches

Adams K, Jennison V. What we do not know about police use of Tasers (TM). *Policing*. 2007; 30:447-65.

Azadani PN, Tseng ZH, Ermakov S, Marcus GM, Lee BK. Funding source and author affiliation in TASER research are strongly associated with a conclusion of device safety. *American heart journal*. 2011; 162:533-7.

Belen E, Tipi FF, Bayyigit A, Helvaci AS. Acute inferior myocardial infarction after electrical weapon exposure: case report and review of the literature. *Turk Kardiyoloji Dernegi arsivi*. 2015; 43:178-81.

Dermengiu D, Hostiuc S, Curca GC. Electroshock weapons: physiologic and pathologic effects - literature review. *Romanian Journal of Legal Medicine*. 2008; 16:187-93.

Dunet B, Erbland A, Abi-Chahla ML, Tournier C, Fabre T. The TASERed finger: A new entity. Case report and review of literature. *Chirurgie de la main*. 2015; 34:145-8.

Hallett N, Duxbury J, McKee T, et al. Taser use on individuals experiencing mental distress: An integrative literature review. *Journal of Psychiatric and Mental Health Nursing*. 2020.

Ideker RE, Dosdall DJ. Can the direct cardiac effects of the electric pulses generated by the TASER X26 cause immediate or delayed sudden cardiac arrest in normal adults? *The American journal of forensic medicine and pat*hology. 2007; 28:195-201.

Jauchem JR. Deaths in custody: are some due to electronic control devices (including TASER devices) or excited delirium? *Journal of forensic and legal medicine*. 2010; 17:1-7.

Jauchem JR. Repeated or long-duration TASER electronic control device exposures: acidemia and lack of respiration. *Forensic science, medicine, and pathology*. 2010; 6:46-53.

Jauchem JR. Pathophysiologic changes due to TASER(R) devices versus excited delirium: potential relevance to deaths-in-custody? *Journal of forensic and legal medicine*. 2011; 18:145-53.

Jauchem JR. Blood lactate concentration after exposure to conducted energy weapons (including TASER(R) devices): is it clinically relevant? *Forensic science, medicine, and pathology*. 2013; 9:386-94.

Jauchem JR. TASER(R) conducted electrical weapons: misconceptions in the scientific/medical and other literature. *Forensic science, medicine, and pathology*. 2015; 11:53-64.

Jauchem JR. Exposures to conducted electrical weapons (including TASER(R) devices): how many and for how long are acceptable? *Journal of forensic sciences*. 2015; 60 Suppl 1:S116-29.

Kroll MW. Physiology and pathology of TASER electronic control devices. *Journal of forensic and legal medicine*. 2009; 16:173-7.

Kunz SN, Grove N, Fischer F. Acute pathophysiological influences of conducted electrical weapons in humans: A review of current literature. *Forensic science international.* 2012; 221:1-4.

Kunz SN, Zinka B, Fieseler S, Graw M, Peschel O. Functioning and effectiveness of electronic control devices such as the TASER(R) M- and X-series: a review of the current literature. *Journal of forensic sciences*. 2012; 57:1591-4.

Kunz SN, Adamec J. A comparative brief on conducted electrical weapon safety. *Wiener Medizinische Wochenschrift.* 2018:1-8.

Kunz SN, Calkins H, Adamec J, Kroll MW. Cardiac and skeletal muscle effects of electrical weapons : A review of human and animal studies. *Forensic science, medicine, and pathology*. 2018; 14:358-66.

Kunz SN, Calkins HG, Adamec J, Kroll MW. Adrenergic and metabolic effects of electrical weapons: review and meta-analysis of human data. *International journal of legal medicine*. 2018; 132:1469-75.

Laub J. Study of deaths following electro muscular disruption. *National Institute of Justice*. 2011 May (report).

Nanthakumar K, Massé S, Umapathy K, Dorian P, Sevaptsidis E, Waxman M. Cardiac stimulation with high voltage discharge from stun guns. *CMAJ*. 2008; 178:1451-7.

Nugent K, Bagdure S, Otahbachi M, Cevik C. Conductive energy devices: a review of use and deaths in the United States. *Journal of investigative medicine*. 2011; 59:1203-10.

© 2021 Baliatsas C et al. *JAMA Network Open.*

O'Brien AJ, Thom K. Police use of TASER devices in mental health emergencies: A review. *International Journal of Law and Psychiatry*. 2014; 37:420-6.

Pasquier M, Carron PN, Vallotton L, Yersin B. Electronic control device exposure: a review of morbidity and mortality. *Annals of emergency medicine*. 2011; 58:178-88.

Rich B, Brophy JM. Estimating the Risk of Cardiac Mortality After Exposure to Conducted Energy Weapons. *Canadian Journal of Cardiology*. 2015; 31:1439-46.

Robb M, Close B, Furyk J, Aitken P. Review article: Emergency Department implications of the TASER. *Emergency medicine Australasia*. 2009; 21:250-8.

Schwartz M, Carron PN, Yersin B, Pasquier M. Health Risks Concerning Electronic Control Devices. *Annales Francaises De Medecine D Urgence*. 2015; 5:30-6.

Vilke GM, Chan TC. Less lethal technology: medical issues. *Policing*. 2007; 30:341-57.

Vilke GM, Bozeman WP, Chan TC. Emergency department evaluation after conducted energy weapon use: review of the literature for the clinician. *The Journal of emergency medicine*. 2011; 40:598-604.

Vilke G, Chan T, Bozeman WP, Emergency department evaluation after conducted energy weapon use: review of the literature for the clinician. *J Emerg Med*. 2019;57(5):740-746.

© 2021 Baliatsas C et al. *JAMA Network Open.*

eAppendix 5. Studies on reported associations between death incidents and Taser

Feldman JM, Gruskin S, Coull BA, Krieger N. Quantifying underreporting of law-enforcement-related deaths in United States vital statistics and news-media-based data sources: A capture–recapture analysis. *PLoS medicine*. 2017; 14.

Hall C, Votova K, Heyd C, Walker M, MacDonald S, Eramian D, et al. Restraint in police use of force events: examining sudden in custody death for prone and not-prone positions. *Journal of forensic and legal medicine*. 2015; 31:29-35.

Kroll MW, Lakkireddy DR, Stone JR, Luceri RM. TASER electronic control devices and cardiac arrests: coincidental or causal? *Circulation*. 2014; 129:93-100.

Kroll MW, Adamec J, Wetli CV, Williams HE. Fatal traumatic brain injury with electrical weapon falls. *Journal of Forensic and Legal Medicine*. 2016; 43:12-9.

Naunheim RS, Treaster M, Aubin C. Ventricular fibrillation in a man shot with a Taser. *Emergency medicine journal* : EMJ. 2010; 27:645-6.

Strote J, Range Hutson H. Taser use in restraint-related deaths. *Prehospital emergency care*. 2006; 10:447-50.

Swerdlow CD, Fishbein MC, Chaman L, Lakkireddy DR, Tchou P. Presenting rhythm in sudden deaths temporally proximate to discharge of TASER conducted electrical weapons. *Academic emergency medicine*. 2009; 16:726-39.

White MD, Ready J. Examining fatal and nonfatal incidents involving the TASER: Identifying predictors of suspect death reported in the media. *Criminology & Public Policy*. 2009;8:865-91.

White MD, Ready J, Riggs C, Dawes DM, Hinz A, Ho JD. An incident-level profile of TASER device deployments in arrest-related deaths. *Police Quarterly*. 2013; 16:85-112.

Zipes DP. Sudden cardiac arrest and death following application of shocks from a TASER electronic control device. *Circulation*. 2012; 125:2417-22.

Zipes DP. TASER electronic control devices can cause cardiac arrest in humans. *Circulation*. 2014; 129:101-11.

© 2021 Baliatsas C et al. *JAMA Network Open.*

eAppendix 6. Data base Searches
Web of Science search

# 37	675	#35 NOT #36<br>*Indexes=SCI-EXPANDED, SSCI, A&HCI, BKCI-S, BKCI-SSH, ESCI Timespan=All years*
# 36	1,018,866	**TOPIC:** (animal*)<br>*Indexes=SCI-EXPANDED, SSCI, A&HCI, BKCI-S, BKCI-SSH, ESCI Timespan=All years*
# 35	714	#32 OR #31 OR #30 OR #29 OR #28 OR #27 OR #26 OR #25 OR #24 OR #23 OR #22 OR #21 OR #20 OR #19 OR #18 OR #17 OR #16 OR #15 OR #14 OR #13 OR #12 OR #11 OR #10 OR #9 OR #8 OR #7 OR #6 OR #5 OR #4 OR #3 OR #2 OR #1<br>**Refined by: PUBLICATION YEARS:** (2019 OR 2011 OR 2003 OR 2018 OR 2010 OR 2002 OR 2017 OR 2009 OR 2001 OR 2016 OR 2008 OR 2000 OR 2015 OR 2007 OR 2014 OR 2006 OR 2013 OR 2005 OR 2012 OR 2004) AND **DOCUMENT TYPES:** (ARTICLE OR REVIEW)<br>*Indexes=SCI-EXPANDED, SSCI, A&HCI, BKCI-S, BKCI-SSH, ESCI Timespan=All years*
# 34	908	#32 OR #31 OR #30 OR #29 OR #28 OR #27 OR #26 OR #25 OR #24 OR #23 OR #22 OR #21 OR #20 OR #19 OR #18 OR #17 OR #16 OR #15 OR #14 OR #13 OR #12 OR #11 OR #10 OR #9 OR #8 OR #7 OR #6 OR #5 OR #4 OR #3 OR #2 OR #1<br>**Refined by: PUBLICATION YEARS:** (2019 OR 2011 OR 2003 OR 2018 OR 2010 OR 2002 OR 2017 OR 2009 OR 2001 OR 2016 OR 2008 OR 2000 OR 2015 OR 2007 OR 2014 OR 2006 OR 2013 OR 2005 OR 2012 OR 2004)<br>*Indexes=SCI-EXPANDED, SSCI, A&HCI, BKCI-S, BKCI-SSH, ESCI Timespan=All years*
# 33	979	#32 OR #31 OR #30 OR #29 OR #28 OR #27 OR #26 OR #25 OR #24 OR #23 OR #22 OR #21 OR #20 OR #19 OR #18 OR #17 OR #16 OR #15 OR #14 OR #13 OR #12 OR #11 OR #10 OR #9 OR #8 OR #7 OR #6 OR #5 OR #4 OR #3 OR #2 OR #1<br>*Indexes=SCI-EXPANDED, SSCI, A&HCI, BKCI-S, BKCI-SSH, ESCI Timespan=All years*
# 32	13	**TOPIC:** ("electric weapon*")<br>*Indexes=SCI-EXPANDED, SSCI, A&HCI, BKCI-S, BKCI-SSH, ESCI Timespan=All years*
# 31	2	**TOPIC:** ("electrical shock device*")<br>*Indexes=SCI-EXPANDED, SSCI, A&HCI, BKCI-S, BKCI-SSH, ESCI Timespan=All years*
# 30	15	**TOPIC :** ("range projectile*")<br>*Indexes=SCI-EXPANDED, SSCI, A&HCI, BKCI-S, BKCI-SSH, ESCI Timespan=All years*
# 29	2	**TOPIC:** ("electric shock weapon*")<br>*Indexes=SCI-EXPANDED, SSCI, A&HCI, BKCI-S, BKCI-SSH, ESCI Timespan=All years*
# 28	5	**TOPIC:** ("electric shock device*")<br>*Indexes=SCI-EXPANDED, SSCI, A&HCI, BKCI-S, BKCI-SSH, ESCI Timespan=All years*
# 27	127	**TOPIC:** ("electrical weapon*")<br>*Indexes=SCI-EXPANDED, SSCI, A&HCI, BKCI-S, BKCI-SSH, ESCI Timespan=All years*
# 26	22	**TOPIC:** ("electronic weapon*")<br>*Indexes=SCI-EXPANDED, SSCI, A&HCI, BKCI-S, BKCI-SSH, ESCI Timespan=All years*
# 25	14	**TOPIC:** ("conductive energy device*")<br>*Indexes=SCI-EXPANDED, SSCI, A&HCI, BKCI-S, BKCI-SSH, ESCI Timespan=All years*
# 24	6	**TOPIC:** ("electromuscular disruption*")<br>*Indexes=SCI-EXPANDED, SSCI, A&HCI, BKCI-S, BKCI-SSH, ESCI Timespan=All years*
# 23	7	**TOPIC:** ("neuromuscular incapacitation*")<br>*Indexes=SCI-EXPANDED, SSCI, A&HCI, BKCI-S, BKCI-SSH, ESCI Timespan=All years*
# 22	9	**TOPIC:** ("incapacitation device*")<br>*Indexes=SCI-EXPANDED, SSCI, A&HCI, BKCI-S, BKCI-SSH, ESCI Timespan=All years*
# 21	17	**TOPIC:** ("range weapon*")<br>*Indexes=SCI-EXPANDED, SSCI, A&HCI, BKCI-S, BKCI-SSH, ESCI Timespan=All years*
# 20	3	**TOPIC:** ("conductive electronic device*")<br>*Indexes=SCI-EXPANDED, SSCI, A&HCI, BKCI-S, BKCI-SSH, ESCI Timespan=All years*
# 19	140	**TOPIC:** ("electronic control device*")<br>*Indexes=SCI-EXPANDED, SSCI, A&HCI, BKCI-S, BKCI-SSH, ESCI Timespan=All years*
# 18	207	**TOPIC:** (CEW)<br>*Indexes=SCI-EXPANDED, SSCI, A&HCI, BKCI-S, BKCI-SSH, ESCI Timespan=All years*

© 2021 Baliatsas C et al. *JAMA Network Open.*

# 17	**37**	**TOPIC:** ("conducted energy weapon*") *Indexes=SCI-EXPANDED, SSCI, A&HCI, BKCI-S, BKCI-SSH, ESCI Timespan=All years*
# 16	**30**	**TOPIC:** ("conducted energy device*") *Indexes=SCI-EXPANDED, SSCI, A&HCI, BKCI-S, BKCI-SSH, ESCI Timespan=All years*
# 15	**7**	**TOPIC:** ("impact weapon*") *Indexes=SCI-EXPANDED, SSCI, A&HCI, BKCI-S, BKCI-SSH, ESCI Timespan=All years*
# 14	**26**	**TOPIC:** ("less-lethal weapon*") *Indexes=SCI-EXPANDED, SSCI, A&HCI, BKCI-S, BKCI-SSH, ESCI Timespan=All years*
# 13	**3**	**TOPIC:** ("non-lethal device*") *Indexes=SCI-EXPANDED, SSCI, A&HCI, BKCI-S, BKCI-SSH, ESCI Timespan=All years*
# 12	**71**	**TOPIC:** ("non-lethal weapon*") *Indexes=SCI-EXPANDED, SSCI, A&HCI, BKCI-S, BKCI-SSH, ESCI Timespan=All years*
# 11	**122**	**TOPIC:** ("energy weapon*") *Indexes=SCI-EXPANDED, SSCI, A&HCI, BKCI-S, BKCI-SSH, ESCI Timespan=All years*
# 10	**110**	**TOPIC:** ("conducted electrical weapon*") *Indexes=SCI-EXPANDED, SSCI, A&HCI, BKCI-S, BKCI-SSH, ESCI Timespan=All years*
# 9	**138**	**TOPIC:** (CEDs) *Indexes=SCI-EXPANDED, SSCI, A&HCI, BKCI-S, BKCI-SSH, ESCI Timespan=All years*
# 8	**2**	**TOPIC:** ("conducted electrical device*") *Indexes=SCI-EXPANDED, SSCI, A&HCI, BKCI-S, BKCI-SSH, ESCI Timespan=All years*
# 7	**3**	**TOPIC:** ("electroshock weapon*") *Indexes=SCI-EXPANDED, SSCI, A&HCI, BKCI-S, BKCI-SSH, ESCI Timespan=All years*
# 6	**1**	**TOPIC:** ("electronic control weapon*") *Indexes=SCI-EXPANDED, SSCI, A&HCI, BKCI-S, BKCI-SSH, ESCI Timespan=All years*
# 5	**140**	**TOPIC:** ("electronic control device*") *Indexes=SCI-EXPANDED, SSCI, A&HCI, BKCI-S, BKCI-SSH, ESCI Timespan=All years*
# 4	**1**	**TOPIC:** ("electronic control weapon*") *Indexes=SCI-EXPANDED, SSCI, A&HCI, BKCI-S, BKCI-SSH, ESCI Timespan=All years*
# 3	**3**	**TOPIC:** ("electroshock weapon*") *Indexes=SCI-EXPANDED, SSCI, A&HCI, BKCI-S, BKCI-SSH, ESCI Timespan=All years*
# 2	**364**	**TOPIC:** ("Taser®") *Indexes=SCI-EXPANDED, SSCI, A&HCI, BKCI-S, BKCI-SSH, ESCI Timespan=All years*
# 1	**409**	**TOPIC:** (taser*) *Indexes=SCI-EXPANDED, SSCI, A&HCI, BKCI-S, BKCI-SSH, ESCI Timespan=All years*

© 2021 Baliatsas C et al. *JAMA Network Open.*

Embase
Session Results
....

No.	Query	Results	Date
#28.	#25 AND 'human'/de AND (2000:py OR 2001:py OR 2002:py OR 2003:py OR 2004:py OR 2005:py OR 2006:py OR 2007:py OR 2008:py OR 2009:py OR 2010:py OR 2011:py OR 2012:py OR 2013:py OR 2014:py OR 2015:py OR 2016:py OR 2017:py OR 2018:py OR 2019:py) AND ('Article'/it OR 'Article in Press'/it OR 'Review'/it OR 'Short Survey'/it)	433	25 Apr 2019
#27.	#25 AND 'human'/de AND (2000:py OR 2001:py OR 2002:py OR 2003:py OR 2004:py OR 2005:py OR 2006:py OR 2007:py OR 2008:py OR 2009:py OR 2010:py OR 2011:py OR 2012:py OR 2013:py OR 2014:py OR 2015:py OR 2016:py OR 2017:py OR 2018:py OR 2019:py)	601	25 Apr 2019
#26.	#25 AND 'human'/de	630	25 Apr 2019
#25.	#1 OR #2 OR #3 OR #4 OR #5 OR #6 OR #7 OR #8 OR #9 OR #10 OR #11 OR #12 OR #13 OR #14 OR #15 OR #16 OR #17 OR #18 OR #19 OR #20 OR #21 OR #22 OR #23 OR #24	827	25 Apr 2019
#24.	'stun gun':ti,ab,kw	40	25 Apr 2019
#23.	'electric weapon*':ti,ab,kw	2	25 Apr 2019
#22.	'electric shock device*':ti,ab,kw	8	25 Apr 2019
#21.	'electric shock weapon*':ti,ab,kw	1	25 Apr 2019
#20.	'incapacitation device*':ti,ab,kw	17	25 Apr 2019
#19.	'range projectile*':ti,ab,kw	7	25 Apr 2019
#18.	'electrical weapon*':ti,ab,kw	116	25 Apr 2019
#17.	'electronic weapon*':ti,ab,kw	13	25 Apr 2019
#16.	'conductive energy device*':ti,ab,kw	11	25 Apr 2019
#15.	'electromuscular disruption*':ti,ab,kw	4	25 Apr 2019
#14.	'neuromuscular incapacitation*':ti,ab,kw	17	25 Apr 2019
#13.	'electronic control device*':ti,ab,kw	102	25 Apr 2019
#12.	cew:ti,ab,kw	154	25 Apr 2019
#11.	'conducted energy weapon*':ti,ab,kw	33	25 Apr 2019
#10.	'impact weapon*':ti,ab,kw	7	25 Apr 2019
#9.	'conducted energy device*':ti,ab,kw	11	25 Apr 2019
#8.	'less-lethal weapon*':ti,ab,kw	30	25 Apr 2019

© 2021 Baliatsas C et al. *JAMA Network Open.*

```
#7.  'non-lethal weapon*':ti,ab,kw                                    46  25
Apr 2019
#6.  'energy weapon*':ti,ab,kw                                        53  25
Apr 2019
#5.  'conducted electrical weapon*':ti,ab,kw                         105  25
Apr 2019
#4.  'electroshock weapon*':ti,ab,kw                                   4  25
Apr 2019
#3.  ceds:ti,ab,kw                                                   117  25
Apr 2019
#2.  taser®:ti,ab,kw                                                 389  25
Apr 2019
#1.  'taser'/exp OR taser                                            486  25
Apr 2019
.......................................................
```

© 2021 Baliatsas C et al. *JAMA Network Open.*

Search	Add to builder	Query	Items found
#33	Add	Search **(((((((((((((((((((((((((((taser*[Title/Abstract]) OR "Taser®"[Title/Abstract]) OR CEDs[Title/Abstract]) OR electroshock weapon*[Title/Abstract]) OR conducted electrical weapon*[Title/Abstract]) OR energy weapon*[Title/Abstract]) OR non-lethal weapon*[Title/Abstract]) OR less-lethal weapon*[Title/Abstract]) OR conducted energy device*[Title/Abstract]) OR impact weapon*[Title/Abstract]) OR conducted energy weapon*[Title/Abstract]) OR CEW[Title/Abstract]) OR electronic control device*[Title/Abstract]) OR neuromuscular incapacitation*[Title/Abstract]) OR electromuscular disruption*[Title/Abstract]) OR conductive energy device*[Title/Abstract]) OR electronic weapon*[Title/Abstract]) OR electrical weapon*[Title/Abstract]) OR extended range projectile*[Title/Abstract]) OR incapacitation device*[Title/Abstract]) OR electric shock weapon*[Title/Abstract]) OR electrical shock weapon*[Title/Abstract]) OR electric shock device*[Title/Abstract]) OR electrical shock device*[Title/Abstract]) OR electric weapon*[Title/Abstract]) AND ("2000/01/01"[PDat] : "2019/12/31"[PDat]) AND Humans[Mesh])) OR stun gun*[Title/Abstract]** Filters: **Publication date from 2000/01/01 to 2019/12/31; Humans**	414
#32	Add	Search **((((((((((((((((((((((((taser*[Title/Abstract]) OR "Taser®"[Title/Abstract]) OR CEDs[Title/Abstract]) OR electroshock weapon*[Title/Abstract]) OR conducted electrical weapon*[Title/Abstract]) OR energy weapon*[Title/Abstract]) OR non-lethal weapon*[Title/Abstract]) OR less-lethal weapon*[Title/Abstract]) OR conducted energy device*[Title/Abstract]) OR impact weapon*[Title/Abstract]) OR conducted energy weapon*[Title/Abstract]) OR CEW[Title/Abstract]) OR electronic control device*[Title/Abstract]) OR neuromuscular incapacitation*[Title/Abstract]) OR electromuscular disruption*[Title/Abstract]) OR conductive energy device*[Title/Abstract]) OR electronic weapon*[Title/Abstract]) OR electrical weapon*[Title/Abstract]) OR extended range projectile*[Title/Abstract]) OR incapacitation device*[Title/Abstract]) OR electric shock weapon*[Title/Abstract]) OR electrical shock weapon*[Title/Abstract]) OR electric shock device*[Title/Abstract]) OR electrical shock device*[Title/Abstract]) OR electric weapon*[Title/Abstract]** Filters: **Publication date from 2000/01/01 to 2019/12/31; Humans**	399

© 2021 Baliatsas C et al. *JAMA Network Open.*

Search	Add to builder	Query	Items found
#31	Add	Search **((((((((((((((((((((((((taser*[Title/Abstract]) OR "Taser®"[Title/Abstract]) OR CEDs[Title/Abstract]) OR electroshock weapon*[Title/Abstract]) OR conducted electrical weapon*[Title/Abstract]) OR energy weapon*[Title/Abstract]) OR non-lethal weapon*[Title/Abstract]) OR less-lethal weapon*[Title/Abstract]) OR conducted energy device*[Title/Abstract]) OR impact weapon*[Title/Abstract]) OR conducted energy weapon*[Title/Abstract]) OR CEW[Title/Abstract]) OR electronic control device*[Title/Abstract]) OR neuromuscular incapacitation*[Title/Abstract]) OR electromuscular disruption*[Title/Abstract]) OR conductive energy device*[Title/Abstract]) OR electronic weapon*[Title/Abstract]) OR electrical weapon*[Title/Abstract]) OR extended range projectile*[Title/Abstract]) OR incapacitation device*[Title/Abstract]) OR electric shock weapon*[Title/Abstract]) OR electrical shock weapon*[Title/Abstract]) OR electric shock device*[Title/Abstract]) OR electrical shock device*[Title/Abstract]) OR electric weapon*[Title/Abstract]** Filters: **Publication date from 2003/01/01 to 2019/12/31; Humans**	387
#30	Add	Search **((((((((((((((((((((((((taser*[Title/Abstract]) OR "Taser®"[Title/Abstract]) OR CEDs[Title/Abstract]) OR electroshock weapon*[Title/Abstract]) OR conducted electrical weapon*[Title/Abstract]) OR energy weapon*[Title/Abstract]) OR non-lethal weapon*[Title/Abstract]) OR less-lethal weapon*[Title/Abstract]) OR conducted energy device*[Title/Abstract]) OR impact weapon*[Title/Abstract]) OR conducted energy weapon*[Title/Abstract]) OR CEW[Title/Abstract]) OR electronic control device*[Title/Abstract]) OR neuromuscular incapacitation*[Title/Abstract]) OR electromuscular disruption*[Title/Abstract]) OR conductive energy device*[Title/Abstract]) OR electronic weapon*[Title/Abstract]) OR electrical weapon*[Title/Abstract]) OR extended range projectile*[Title/Abstract]) OR incapacitation device*[Title/Abstract]) OR electric shock weapon*[Title/Abstract]) OR electrical shock weapon*[Title/Abstract]) OR electric shock device*[Title/Abstract]) OR electrical shock device*[Title/Abstract]) OR electric weapon*[Title/Abstract]** Filters: **Publication date from 2003/01/01 to 2019/12/31**	551
#29	Add	Search **((((((((((((((((((((((((taser*[Title/Abstract]) OR "Taser®"[Title/Abstract]) OR CEDs[Title/Abstract]) OR electroshock weapon*[Title/Abstract]) OR conducted electrical weapon*[Title/Abstract]) OR energy weapon*[Title/Abstract]) OR non-lethal weapon*[Title/Abstract]) OR less-lethal weapon*[Title/Abstract]) OR conducted energy device*[Title/Abstract]) OR impact weapon*[Title/Abstract]) OR conducted energy weapon*[Title/Abstract]) OR CEW[Title/Abstract]) OR electronic control device*[Title/Abstract]) OR neuromuscular incapacitation*[Title/Abstract]) OR electromuscular disruption*[Title/Abstract]) OR conductive energy device*[Title/Abstract]) OR electronic weapon*[Title/Abstract]) OR electrical weapon*[Title/Abstract]) OR extended range projectile*[Title/Abstract]) OR incapacitation device*[Title/Abstract]) OR electric shock weapon*[Title/Abstract]) OR electrical shock weapon*[Title/Abstract]) OR electric shock device*[Title/Abstract]) OR electrical shock device*[Title/Abstract]) OR electric weapon*[Title/Abstract]** Filters: **Humans**	434

Search	Add to builder	Query	Items found
#28	Add	Search **((((((((((((((((((((taser*[Title/Abstract]) OR "Taser®"[Title/Abstract]) OR CEDs[Title/Abstract]) OR electroshock weapon*[Title/Abstract]) OR conducted electrical weapon*[Title/Abstract]) OR energy weapon*[Title/Abstract]) OR non-lethal weapon*[Title/Abstract]) OR less-lethal weapon*[Title/Abstract]) OR conducted energy device*[Title/Abstract]) OR impact weapon*[Title/Abstract]) OR conducted energy weapon*[Title/Abstract]) OR CEW[Title/Abstract]) OR electronic control device*[Title/Abstract]) OR neuromuscular incapacitation*[Title/Abstract]) OR electromuscular disruption*[Title/Abstract]) OR conductive energy device*[Title/Abstract]) OR electronic weapon*[Title/Abstract]) OR electrical weapon*[Title/Abstract]) OR extended range projectile*[Title/Abstract]) OR incapacitation device*[Title/Abstract]) OR electric shock weapon*[Title/Abstract]) OR electrical shock weapon*[Title/Abstract]) OR electric shock device*[Title/Abstract]) OR electrical shock device*[Title/Abstract]) OR electric weapon*[Title/Abstract]**	614
#27	Add	Search **electric weapon*[Title/Abstract]**	106
#26	Add	Search **electrical shockdevice*[Title/Abstract]**	2
#25	Add	Search **electric shock device*[Title/Abstract]**	3
#24	Add	Search **electrical shockweapon*[Title/Abstract]**	2
#23	Add	Search **electric shock weapon*[Title/Abstract]**	2
#22	Add	Search **incapacitation device*[Title/Abstract]**	12
#21	Add	Search **range projectile*[Title/Abstract]**	293
#20	Add	Search **extendedrangeprojectile*[Title/Abstract]**	8
#19	Add	Search **electrical weapon*[Title/Abstract]**	92
#18	Add	Search **electronic weapon*[Title/Abstract]**	7
#17	Add	Search **conductive energydevice*[Title/Abstract]**	5
#16	Add	Search **electromuscular disruption*[Title/Abstract]**	2
#15	Add	Search **neuromuscular incapacitation*[Title/Abstract]**	10
#14	Add	Search **electroniccontroldevice*[Title/Abstract]**	61
#13	Add	Search **CEW[Title/Abstract]**	127
#12	Add	Search **conducted energyweapon*[Title/Abstract]**	22
#11	Add	Search **impact weapon*[Title/Abstract]**	5
#10	Add	Search **conducted energydevice*[Title/Abstract]**	4
#9	Add	Search **less-lethal weapon*[Title/Abstract]**	19
#8	Add	Search **non-lethal weapon*[Title/Abstract]**	35
#7	Add	Search **energy weapon*[Title/Abstract]**	38
#6	Add	Search **conducted electrical weapon*[Title/Abstract]**	80
#5	Add	Search **electroshock weapon*[Title/Abstract]**	64
#4	Add	Search **CEDs[Title/Abstract]**	103
#3	Add	Search **"Taser®"[Title/Abstract]**	51
#2	Add	Search **taser*[Title/Abstract]**	295

© 2021 Baliatsas C et al. *JAMA Network Open.*

#	Query	Limiters/Expanders	Results
S35	S1 OR S2 OR S3 OR S5 OR S7 OR S9 OR S10 OR S11 OR S12 OR S13 OR S14 OR S15 OR S16 OR S17 OR S18 OR S19 OR S22 OR [illegible]	Limiters - Publication Year: 2000-2019 Narrow by Population: - human Search modes - Boolean/Phrase	171
S34	S1 OR S2 OR S3 OR S5 OR S7 OR S9 OR S10 OR S11 OR S12 OR S13 OR S14 OR S15 OR S16 OR S17 OR S18 OR S19 OR S22 OR [illegible]	Limiters - Publication Year: 2000-2019 Search modes - Boolean/Phrase	203
S33	S1 OR S2 OR S3 OR S5 OR S7 OR S9 OR S10 OR S11 OR S12 OR S13 OR S14 OR S15 OR S16 OR S17 OR S18 OR S19 OR S22 OR S25 OR S27	Search modes - Boolean/Phrase	226
S32	"electric weapon*"	Search modes - Boolean/Phrase	0
S31	"electrical shock device*"	Search modes - Boolean/Phrase	0

© 2021 Baliatsas C et al. *JAMA Network Open.*

S30	"range projectile*"	Search modes - Boolean/Phrase	0
S29	"electric shock weapon*"	Search modes - Boolean/Phrase	0
S28	"electric shock device*"	Search modes - Boolean/Phrase	0
S27	"electrical weapon*"	Search modes - Boolean/Phrase	1
S26	"electronic weapon*"	Search modes - Boolean/Phrase	0
S25	"conductive energy device*"	Search modes - Boolean/Phrase	2
S24	"electromuscular disruption*"	Search modes - Boolean/Phrase	0
S23	"neuromuscular incapacitation*"	Search modes - Boolean/Phrase	0
S22	"incapacitation device*"	Search modes - Boolean/Phrase	2
S21	"range weapon*"	Search modes - Boolean/Phrase	0

© 2021 Baliatsas C et al. *JAMA Network Open.*

S20	"conductive electronic device*"	Search modes - Boolean/Phrase	0
S19	"electronic control device*"	Search modes - Boolean/Phrase	3
S18	CEW	Search modes - Boolean/Phrase	21
S17	"conducted energy weapon*"	Search modes - Boolean/Phrase	1
S16	"conducted energy device*"	Search modes - Boolean/Phrase	9
S15	"impact weapon*"	Search modes - Boolean/Phrase	1
S14	"less-lethal weapon*"	Search modes - Boolean/Phrase	5
S13	"non-lethal device*"	Search modes - Boolean/Phrase	1
S12	"non-lethal weapon*"	Search modes - Boolean/Phrase	4
S11	"energy weapon*"	Search modes - Boolean/Phrase	4

© 2021 Baliatsas C et al. *JAMA Network Open.*

S10	"conducted electrical weapon*"	Search modes - Boolean/Phrase	1
S9	CEDs	Search modes - Boolean/Phrase	152
S8	"conducted electrical device*"	Search modes - Boolean/Phrase	0
S7	"electroshock weapon*"	Search modes - Boolean/Phrase	2
S6	"electronic control weapon*"	Search modes - Boolean/Phrase	0
S5	"electronic control device*"	Search modes - Boolean/Phrase	3
S4	"electronic control weapon*"	Search modes - Boolean/Phrase	0
S3	"electroshock weapon*"	Search modes - Boolean/Phrase	2
S2	"Taser®"	Search modes - Boolean/Phrase	39
S1	taser*	Search modes - Boolean/Phrase	48

© 2021 Baliatsas C et al. *JAMA Network Open.*

Search Name: taser in Title Abstract Keyword (Word variations have been searched)
Last Saved:27/04/2019 16:53:35

ID Search
#1 taser:ti,ab,kw with Cochrane Library publication date Between Jan 2000 and Dec 2019, in Cochrane Reviews, Cochrane Protocols, Trials (Word variations have been searched) 12

© 2021 Baliatsas C et al. *JAMA Network Open.*

Search Name: Cochrane search (Update)

Date Run: 24/04/2020 13:04:18

Comment:

ID	Search	Hits
#1	(Taser):ti,ab,kw (Word variations have been searched) with Publication Year from 2019 to 2020, with Cochrane Library publication date Between Mar 2019 and Dec 2020, in Trials	6

© 2021 Baliatsas C et al. *JAMA Network Open.*

Web of Science search (Update)

# 37	86	#35 NOT #36<br>*Indexes=SCI-EXPANDED, SSCI, A&HCI, BKCI-S, BKCI-SSH, ESCI Timespan=All years*
# 36	1,083,380	**TOPIC:** (animal*)<br>*Indexes=SCI-EXPANDED, SSCI, A&HCI, BKCI-S, BKCI-SSH, ESCI Timespan=All years*
# 35	89	#32 OR #31 OR #30 OR #29 OR #28 OR #27 OR #26 OR #25 OR #24 OR #23 OR #22 OR #21 OR #20 OR #19 OR #18 OR #17 OR #16 OR #15 OR #14 OR #13 OR #12 OR #11 OR #10 OR #9 OR #8 OR #7 OR #6 OR #5 OR #4 OR #3 OR #2 OR #1<br>**Refined by: PUBLICATION YEARS:** (2020 OR 2019) AND **DOCUMENT TYPES:** (ARTICLE OR REVIEW)<br>*Indexes=SCI-EXPANDED, SSCI, A&HCI, BKCI-S, BKCI-SSH, ESCI Timespan=All years*
# 34	95	#32 OR #31 OR #30 OR #29 OR #28 OR #27 OR #26 OR #25 OR #24 OR #23 OR #22 OR #21 OR #20 OR #19 OR #18 OR #17 OR #16 OR #15 OR #14 OR #13 OR #12 OR #11 OR #10 OR #9 OR #8 OR #7 OR #6 OR #5 OR #4 OR #3 OR #2 OR #1<br>**Refined by: PUBLICATION YEARS:** (2020 OR 2019)<br>*Indexes=SCI-EXPANDED, SSCI, A&HCI, BKCI-S, BKCI-SSH, ESCI Timespan=All years*
# 33	1,059	#32 OR #31 OR #30 OR #29 OR #28 OR #27 OR #26 OR #25 OR #24 OR #23 OR #22 OR #21 OR #20 OR #19 OR #18 OR #17 OR #16 OR #15 OR #14 OR #13 OR #12 OR #11 OR #10 OR #9 OR #8 OR #7 OR #6 OR #5 OR #4 OR #3 OR #2 OR #1<br>*Indexes=SCI-EXPANDED, SSCI, A&HCI, BKCI-S, BKCI-SSH, ESCI Timespan=All years*
# 32	14	**TOPIC:** ("electric weapon*")<br>*Indexes=SCI-EXPANDED, SSCI, A&HCI, BKCI-S, BKCI-SSH, ESCI Timespan=All years*
# 31	2	**TOPIC:** ("electrical shock device*")<br>*Indexes=SCI-EXPANDED, SSCI, A&HCI, BKCI-S, BKCI-SSH, ESCI Timespan=All years*
# 30	16	**TOPIC:** ("range projectile*")<br>*Indexes=SCI-EXPANDED, SSCI, A&HCI, BKCI-S, BKCI-SSH, ESCI Timespan=All years*
# 29	3	**TOPIC:** ("electric shock weapon*")<br>*Indexes=SCI-EXPANDED, SSCI, A&HCI, BKCI-S, BKCI-SSH, ESCI Timespan=All years*
# 28	5	**TOPIC:** ("electric shock device*")<br>*Indexes=SCI-EXPANDED, SSCI, A&HCI, BKCI-S, BKCI-SSH, ESCI Timespan=All years*
# 27	126	**TOPIC:** ("electrical weapon*")<br>*Indexes=SCI-EXPANDED, SSCI, A&HCI, BKCI-S, BKCI-SSH, ESCI Timespan=All years*
# 26	20	**TOPIC:** ("electronic weapon*")<br>*Indexes=SCI-EXPANDED, SSCI, A&HCI, BKCI-S, BKCI-SSH, ESCI Timespan=All years*
# 25	15	**TOPIC:** ("conductive energy device*")<br>*Indexes=SCI-EXPANDED, SSCI, A&HCI, BKCI-S, BKCI-SSH, ESCI Timespan=All years*
# 24	6	**TOPIC:** ("electromuscular disruption*")<br>*Indexes=SCI-EXPANDED, SSCI, A&HCI, BKCI-S, BKCI-SSH, ESCI Timespan=All years*
# 23	7	**TOPIC:** ("neuromuscular incapacitation*")<br>*Indexes=SCI-EXPANDED, SSCI, A&HCI, BKCI-S, BKCI-SSH, ESCI Timespan=All years*
# 22	9	**TOPIC:** ("incapacitation device*")<br>*Indexes=SCI-EXPANDED, SSCI, A&HCI, BKCI-S, BKCI-SSH, ESCI Timespan=All years*
# 21	19	**TOPIC:** ("range weapon*")<br>*Indexes=SCI-EXPANDED, SSCI, A&HCI, BKCI-S, BKCI-SSH, ESCI Timespan=All years*

© 2021 Baliatsas C et al. *JAMA Network Open.*

# 20	4	**TOPIC:** ("conductive electronic device*")<br>*Indexes=SCI-EXPANDED, SSCI, A&HCI, BKCI-S, BKCI-SSH, ESCI Timespan=All years*
# 19	137	**TOPIC:** ("electronic control device*")<br>*Indexes=SCI-EXPANDED, SSCI, A&HCI, BKCI-S, BKCI-SSH, ESCI Timespan=All years*
# 18	229	**TOPIC:** (CEW)<br>*Indexes=SCI-EXPANDED, SSCI, A&HCI, BKCI-S, BKCI-SSH, ESCI Timespan=All years*
# 17	40	**TOPIC:** ("conducted energy weapon*")<br>*Indexes=SCI-EXPANDED, SSCI, A&HCI, BKCI-S, BKCI-SSH, ESCI Timespan=All years*
# 16	34	**TOPIC:** ("conducted energy device*")<br>*Indexes=SCI-EXPANDED, SSCI, A&HCI, BKCI-S, BKCI-SSH, ESCI Timespan=All years*
# 15	10	**TOPIC:** ("impact weapon*")<br>*Indexes=SCI-EXPANDED, SSCI, A&HCI, BKCI-S, BKCI-SSH, ESCI Timespan=All years*
# 14	29	**TOPIC:** ("less-lethal weapon*")<br>*Indexes=SCI-EXPANDED, SSCI, A&HCI, BKCI-S, BKCI-SSH, ESCI Timespan=All years*
# 13	3	**TOPIC:** ("non-lethal device*")<br>*Indexes=SCI-EXPANDED, SSCI, A&HCI, BKCI-S, BKCI-SSH, ESCI Timespan=All years*
# 12	77	**TOPIC:** ("non-lethal weapon*")<br>*Indexes=SCI-EXPANDED, SSCI, A&HCI, BKCI-S, BKCI-SSH, ESCI Timespan=All years*
# 11	133	**TOPIC:** ("energy weapon*")<br>*Indexes=SCI-EXPANDED, SSCI, A&HCI, BKCI-S, BKCI-SSH, ESCI Timespan=All years*
# 10	110	**TOPIC:** ("conducted electrical weapon*")<br>*Indexes=SCI-EXPANDED, SSCI, A&HCI, BKCI-S, BKCI-SSH, ESCI Timespan=All years*
# 9	163	**TOPIC:** (CEDs)<br>*Indexes=SCI-EXPANDED, SSCI, A&HCI, BKCI-S, BKCI-SSH, ESCI Timespan=All years*
# 8	2	**TOPIC:** ("conducted electrical device*")<br>*Indexes=SCI-EXPANDED, SSCI, A&HCI, BKCI-S, BKCI-SSH, ESCI Timespan=All years*
# 7	3	**TOPIC:** ("electroshock weapon*")<br>*Indexes=SCI-EXPANDED, SSCI, A&HCI, BKCI-S, BKCI-SSH, ESCI Timespan=All years*
# 6	1	**TOPIC:** ("electronic control weapon*")<br>*Indexes=SCI-EXPANDED, SSCI, A&HCI, BKCI-S, BKCI-SSH, ESCI Timespan=All years*
# 5	137	**TOPIC:** ("electronic control device*")<br>*Indexes=SCI-EXPANDED, SSCI, A&HCI, BKCI-S, BKCI-SSH, ESCI Timespan=All years*
# 4	1	**TOPIC:** ("electronic control weapon*")<br>*Indexes=SCI-EXPANDED, SSCI, A&HCI, BKCI-S, BKCI-SSH, ESCI Timespan=All years*
# 3	3	**TOPIC:** ("electroshock weapon*")<br>*Indexes=SCI-EXPANDED, SSCI, A&HCI, BKCI-S, BKCI-SSH, ESCI Timespan=All years*
# 2	377	**TOPIC:** ("Taser®")<br>*Indexes=SCI-EXPANDED, SSCI, A&HCI, BKCI-S, BKCI-SSH, ESCI Timespan=All years*
# 1	426	**TOPIC:** (taser*)<br>*Indexes=SCI-EXPANDED, SSCI, A&HCI, BKCI-S, BKCI-SSH, ESCI Timespan=All years*

Advanced search - PubMed (Update)

Query

((((((((((((((((((((((((((taser[Title/Abstract] OR tasered[Title/Abstract] OR tasering[Title/Abstract] OR taserk[Title/Abstract] OR taserk1[Title/Abstract] OR taserk2[Title/Abstract] OR taserk3[Title/Abstract] OR taserk4[Title/Abstract] OR taserk5[Title/Abstract] OR taserks[Title/Abstract] OR tasero[Title/Abstract] OR taseros[Title/Abstract] OR tasers[Title/Abstract] OR tasertrade[Title/Abstract] OR taserx26[Title/Abstract]) OR "Taser(R)"[Title/Abstract]) OR CEDs[Title/Abstract]) OR (("electroshock"[MeSH Terms] OR "electroshock"[All Fields]) AND (weapon[Title/Abstract] OR weapon'[Title/Abstract] OR weapon's[Title/Abstract] OR weaponed[Title/Abstract] OR weaponeers[Title/Abstract] OR weaponisation[Title/Abstract] OR weaponise[Title/Abstract] OR weaponised[Title/Abstract] OR weaponised'[Title/Abstract] OR weaponising[Title/Abstract] OR weaponizable[Title/Abstract] OR weaponization[Title/Abstract] OR weaponize[Title/Abstract] OR weaponized[Title/Abstract] OR weaponized'[Title/Abstract] OR weaponizes[Title/Abstract] OR weaponizing[Title/Abstract] OR weaponless[Title/Abstract] OR weaponries[Title/Abstract] OR weaponry[Title/Abstract] OR weaponry's[Title/Abstract] OR weapons[Title/Abstract] OR weapons'[Title/Abstract] OR weapons'laws[Title/Abstract]))) OR (conducted electrical weapon[Title/Abstract] OR conducted electrical weapons[Title/Abstract])) OR (energy weapon[Title/Abstract] OR energy weapons[Title/Abstract])) OR (non lethal weapon[Title/Abstract] OR non lethal weapons[Title/Abstract])) OR (less lethal weapon[Title/Abstract] OR less lethal weapons[Title/Abstract])) OR (conducted energy device[Title/Abstract] OR conducted energy devices[Title/Abstract])) OR impact weapons[Title/Abstract]) OR (conducted energy weapon[Title/Abstract] OR conducted energy weapons[Title/Abstract])) OR CEW[Title/Abstract]) OR (electronic control device[Title/Abstract] OR electronic control devices[Title/Abstract])) OR neuromuscular incapacitation[Title/Abstract]) OR electromuscular disruption[Title/Abstract]) OR conductive energy devices[Title/Abstract]) OR (electronic weapon[Title/Abstract] OR electronic weaponry[Title/Abstract] OR electronic weapons[Title/Abstract])) OR (electrical weapon[Title/Abstract] OR electrical weapons[Title/Abstract])) OR ((extended[All Fields] AND range[All Fields]) AND (projectile[Title/Abstract] OR projectile'[Title/Abstract] OR projectile's[Title/Abstract] OR projectilelike[Title/Abstract] OR projectilerich[Title/Abstract] OR projectiles[Title/Abstract] OR projectiles'[Title/Abstract]))) OR (range[All Fields] AND (projectile[Title/Abstract] OR projectile'[Title/Abstract] OR projectile's[Title/Abstract] OR projectilelike[Title/Abstract] OR projectilerich[Title/Abstract] OR projectiles[Title/Abstract] OR projectiles'[Title/Abstract]))) OR incapacitation devices[Title/Abstract]) OR electric shock weapons[Title/Abstract]) OR (("electricity"[MeSH Terms] OR "electricity"[All Fields] OR "electrical"[All Fields]) AND shock weapons[Title/Abstract])) OR electric shock devices[Title/Abstract]) OR (("electricity"[MeSH Terms] OR "electricity"[All Fields] OR "electrical"[All Fields]) AND (shock device[Title/Abstract] OR shock devices[Title/Abstract]))) OR (("electricity"[MeSH Terms] OR "electricity"[All Fields] OR "electric"[All Fields]) AND (weapon[Title/Abstract] OR weapon'[Title/Abstract] OR weapon's[Title/Abstract] OR weaponed[Title/Abstract] OR weaponeers[Title/Abstract] OR weaponisation[Title/Abstract] OR weaponise[Title/Abstract] OR weaponised[Title/Abstract] OR weaponised'[Title/Abstract] OR

© 2021 Baliatsas C et al. *JAMA Network Open.*

weaponising[Title/Abstract] OR weaponizable[Title/Abstract] OR weaponization[Title/Abstract] OR weaponize[Title/Abstract] OR weaponized[Title/Abstract] OR weaponized'[Title/Abstract] OR weaponizes[Title/Abstract] OR weaponizing[Title/Abstract] OR weaponless[Title/Abstract] OR weaponries[Title/Abstract] OR weaponry[Title/Abstract] OR weaponry's[Title/Abstract] OR weapons[Title/Abstract] OR weapons'[Title/Abstract] OR weapons'laws[Title/Abstract]))) OR (stun gun[Title/Abstract] OR stun guns[Title/Abstract]) AND (("2019/03/01"[PDAT] : "2020/12/31"[PDAT]) AND "humans"[MeSH Terms])

Items found 21

© 2021 Baliatsas C et al. *JAMA Network Open.*

#	Query	Limiters/Expanders	Results
S21	S1 OR S2 OR S3 OR S4	Limiters - Published Date:	3
	OR S5 OR S6 OR S7 OR	20190301-20201231	
	S8 OR S9 OR S10 OR	Expanders - Apply	
	S11 OR S12 OR S13 OR	equivalent subjects	
	S14 OR S15 OR S16 OR	Search modes -	
	S17 OR S18 OR S19	Boolean/Phrase	
S20	S1 OR S2 OR S3 OR S4	Expanders - Apply	233
	OR S5 OR S6 OR S7 OR	equivalent subjects	
	S8 OR S9 OR S10 OR	Search modes -	
	S11 OR S12 OR S13 OR	Boolean/Phrase	
	S14 OR S15 OR S16 OR		
	S17 OR S18 OR S19		
S19	"electrical weapon*"	Expanders - Apply	2
		equivalent subjects	
		Search modes -	
		Boolean/Phrase	
S18	"conductive energy device*"	Expanders - Apply equivalent	2
		Search modes -	
		Boolean/Phrase	
S17	"incapacitation device*"	Expanders - Apply equivalent	2
		Search modes -	
		Boolean/Phrase	
S16	"electronic control device*"	Expanders - Apply equivalent	3
		Search modes -	
		Boolean/Phrase	
S15	CEW	Expanders - Apply	21
		equivalent subjects	
		Search modes -	
		Boolean/Phrase	

© 2021 Baliatsas C et al. *JAMA Network Open.*

S14	"conducted energy weapon*"	Expanders - Apply equivalent subjects Search modes - Boolean/Phrase	2
S13	"conducted energy device*"	Expanders - Apply equivalent subjects Search modes - Boolean/Phrase	9
S12	"impact weapon*"	Expanders - Apply equivalent subjects Search modes - Boolean/Phrase	1
S11	"less-lethal weapon*"	Expanders - Apply equivalent subjects Search modes - Boolean/Phrase	5
S10	"non-lethal device*"	Expanders - Apply equivalent subjects Search modes - Boolean/Phrase	1
S9	"non-lethal weapon*"	Expanders - Apply equivalent subjects Search modes - Boolean/Phrase	4
S8	"energy weapon*"	Expanders - Apply equivalent subjects Search modes - Boolean/Phrase	5
S7	“conducted electrical weapon*”	Expanders - Apply equivalent subjects Search modes - Boolean/Phrase	2
S6	CEDs	Expanders - Apply equivalent subjects Search modes - Boolean/Phrase	154
S5	“electroshock weapon*"	Expanders - Apply equivalent subjects Search modes - Boolean/Phrase	2

© 2021 Baliatsas C et al. *JAMA Network Open.*

S4	"electronic control device*"	Expanders - Apply equivalent subjects Search modes - Boolean/Phrase	3
S3	"electroshock weapon*"	Expanders - Apply equivalent subjects Search modes - Boolean/Phrase	2
S2	"Taser®"	Expanders - Apply equivalent subjects Search modes - Boolean/Phrase	43
S1	Taser*	Expanders - Apply equivalent subjects Search modes - Boolean/Phrase	53

© 2021 Baliatsas C et al. *JAMA Network Open.*
